# White matter microstructural and macrostructural profiles during midlife reveal sex differences between men and women at different menopausal stages

**DOI:** 10.1038/s41598-025-24136-y

**Published:** 2025-11-17

**Authors:** Adam C. Raikes, Jonathan P. Dyke, Matilde Nerattini, Camila Boneu, Trisha Ajila, Francesca Fauci, Michael Battista, Silky Pahlajani, Schantel Williams, Roberta Diaz Brinton, Lisa Mosconi

**Affiliations:** 1 Center for Innovation in Brain Science, University of Arizona, Tucson, AZ 85719 USA; 2 Department of Radiology, Weill Cornell Medicine, New York, NY 10021 USA; 3 Department of Neurology, Weill Cornell Medicine, New York, NY 10021 USA; 4 Department of Pharmacology, University of Arizona, Tucson, AZ 85719 USA; 5 Department of Neurology, University of Arizona, Tucson, AZ 85719 USA

**Keywords:** Magnetic resonance imaging, Brain imaging

## Abstract

Women are at greater lifetime risk for Alzheimer’s disease (AD), potentially due to midlife endocrine transition effects on bioenergetics and gray and white matter volumes. Key to effective neurotransmission is white matter integrity. In this cross-sectional diffusion-weighted imaging analysis, we investigated white matter micro- and macrostructure in 137 cognitively normal, midlife adults, including 34 premenopausal, 39 perimenopausal, 27 postmenopausal women, and 37 men. Compared to men, premenopausal and postmenopausal women exhibited greater fiber density and fiber density-cross-section (FDC) across most tracts as well as higher fractional anisotropy (FA) and lower mean diffusivity in the fornix and corpus callosum, indicating denser, more organized white matter. Perimenopausal women exhibited few differences in fiber cross-section and FDC and lower FA in the bilateral fornix, differing from what was observed in pre- and postmenopausal women. These cross-sectional results suggest menopause stage-dependent sex differences in white matter organization, coinciding with changes in estrogen availability. Importantly, the perimenopause emerges as a critical window of neural reorganization in the female midlife aging brain characterized by temporary convergence toward male-like white matter organization. Future longitudinal analyses are key to identifying women who do or do not revert to a premenopausal profile, which may inform AD risk.

**Supplementary Information:**

The online version contains supplementary material available at 10.1038/s41598-025-24136-y.

## Introduction

Women have a greater prevalence of late-onset Alzheimer’s disease (AD), independent of age and survival rates, with postmenopausal women representing more than 60% of AD cases^1^. AD has a long prodromal period lasting 10–20 years prior to clinical diagnosis. This prodromal phase aligns with the menopausal timeline, suggesting that menopause may play a role in elevated risk in women after accounting for other risk factors, such as age and APOE genotype. This is supported by cross-sectional studies demonstrating increased amyloid-beta and tau pathology, decreased gray matter volume, cerebral glucose dysregulation, and altered mitochondrial function in AD-vulnerable brain regions in peri- and postmenopausal women compared to either premenopausal women or age-matched men^2–7^. Less well-described than these AD-relevant markers is the role of sex and menopause on white matter.

Fractional anisotropy (FA) is the most frequently reported metric of white matter microstructure, with higher values generally associated with greater white matter health. Across the literature, there is substantial variability regarding sex differences in FA, with studies reporting men having higher FA than women^8–11^, women having higher FA than men^12,13^, or limited to no sex differences at all^14,15^. Regions most commonly associated with sex differences include the fornix, corpus callosum, cingulum, corona radiata, and thalamic radiata. Women typically show greater FA in the fornix^2,16–19^ while men often show greater FA in the cingulum and thalamic radiata^2,12,18,20,21^. The corpus callosum exhibits mixed profiles across studies^12,15–17,22,23^. These pathways - particularly the fornix, cingulum, corona radiata and corpus callosum - also exhibit reduced FA in persons with mild cognitive impairment and AD^24–28^. In addition, sex differences are observed in AD, with women having lower FA throughout the white matter compared to men^14,29^. If sex differences exist in the white matter earlier in life and if menopause is a driver of greater AD risk in women, then it is possible that sex differences in white matter microstructure prior to and during the menopausal transition contribute to this risk disparity. Research describing sex differences on white matter microstructure with consideration for menopausal transition status remains limited^2^.

Another understudied area is differences between men and women in white matter macrostructural organization. One method for probing such macrostructural features using diffusion MRI is a “fixel-based” approach^30^. This approach characterizes the fiber orientations present within a voxel, providing measures of both microstructural fiber density (FD; amplitude of the fiber orientation distribution which is linearly proportional to total intra-axonal volume)^31^ and macrostructural fiber-cross section (FC; local volume difference along the cross-sectional area perpendicular to the fixel orientation derived from the spatial warps to template space)^30,32^. This provides complementary information to diffusion MRI tensor metrics, capturing measures sensitive to fiber loss, bundle atrophy, and a combined metric that indexes the simultaneous contributions of density and cross-section (FDC; a macrostructural estimate of total intra-axonal matter computed as the product of FD and FC)^30,32^.

Recent work using this approach has demonstrated reduced FD, FC, and FDC in persons with either mild cognitive impairment or mild amnestic AD compared to cognitively unimpaired individuals^33–35^. These differences occurred primarily in limbic and callosal fiber bundles^33–35^ indicating both fiber loss and bundle atrophy in pathways exhibiting reduced FA^24–28^. However, sex-specific fixel-based analyses in cognitively normal and healthy middle-aged adults are limited^36–38^. Across those studies, sex was a secondary consideration relative to aging effects and the direction of identified sex differences (i.e. women > men) was not clearly described. The absence of more comprehensive evaluations of sex differences in these micro- and macrostructural properties, particularly in midlife, limits our understanding of how sex differences may contribute to disease risk and onset.

Herein we conducted a cross-sectional analysis of sex differences in fixel- and diffusion tensor based white matter properties in cognitively normal men and women in midlife. The primary analyses focused on sex differences in this cohort, with analyses stratified by menopausal status in women (pre-, peri-, and postmenopausal). Given the lack of consensus on the presence, absence, and spatial distribution of sex differences in white matter, we employed a Bayesian hierarchical analysis framework. This approach integrates all of the tracts into a singular random effects model in order to leverage partial pooling and mitigate statistical concerns of multiplicity^39,40^. Outcomes from this analytic approach describe the probability of sex by menopause status differences within the data, enabling detection of both the directionality and strength of evidence for a directional difference. Using this strategy, we aimed to provide a robust framework for identifying cross-sectional sex differences as well as hypothesis-generating data and informative priors for future longitudinal studies.

## Results

### Demographics

After excluding individuals with incomplete datasets, menopausal hormone therapy (MHT) users, and women with a history of oophorectomy and/or hysterectomy, a total of 137 individuals aged 36–65 were included in the analyses. This included 34 pre-, 39 peri-, and 27 postmenopausal women and a total of 37 men. All participants were in good general health. Demographic data are presented in Table 1 and demographics for the menopause stage stratified analyses are presented in Table 2.

**Table 1:** Demographic characteristics across all participants.

	Male *N* = 37	Female *N* = 100
Age^1^	51.35 (7.68)	49.49 (6.20)
Years of education^1^	17.51 (2.02)	17.10 (1.54)
Total cholesterol (mg/dL)^1^	180.81 (39.18)	199.87 (32.27)
BMI^1^	27.29 (4.60)	25.03 (5.53)
Total intracranial volume (cm^3^)^1^	1,624.45 (134.20)	1,411.37 (95.57)
APOE-ε4 carriers^2^	21 (57%)	48 (48%)
Family history of Alzheimer’s^2^	25 (68%)	82 (82%)
History of Hypertension^2^	8 (22%)	6 (6.1%)

^1^ Mean (SD).

^2^ n (%).

**Table 2:** Demographic characteristics for females in each menopausal stage and age-controlled males.

	Male *N* = 18	Female PreM *N* = 34	Male *N* = 21	Female PeriM *N* = 39	Male *N* = 18	Female PostM *N* = 27
Age^1^	45.39 (3.11)	44.06 (3.13)	47.86 (4.04)	49.00 (3.74)	58.17 (3.82)	57.04 (3.95)
Years of education^1^	17.11 (1.84)	17.00 (1.23)	17.33 (1.93)	16.97 (1.77)	18.00 (2.17)	17.41 (1.55)
Total cholesterol (mg/dL)^1^	192.82 (32.88)	190.56 (36.54)	180.30 (44.32)	204.97 (28.20)	164.44 (35.18)	204.41 (30.42)
BMI^1^	27.29 (3.19)	24.19 (4.90)	27.47 (4.16)	26.36 (6.03)	27.29 (5.78)	24.09 (5.28)
Total intracranial volume (cm^3^)^1^	1,631.24 (123.25)	1,417.00 (99.21)	1,631.37 (136.50)	1,414.82 (98.36)	1,618.48 (151.14)	1,399.30 (89.09)
APOE-ε4 carriers^2^	11 (61%)	19 (56%)	9 (43%)	16 (41%)	10 (56%)	13 (48%)
Family history of Alzheimer’s^2^	14 (78%)	25 (74%)	15 (71%)	36 (92%)	10 (56%)	21 (78%)
History of Hypertension^2^	3 (17%)	0 (0%)	4 (19%)	3 (7.7%)	5 (28%)	3 (11%)

^1^ Mean (SD).

^2^ n (%).

PreM: Premenopause; PeriM: Perimenopause; PostM: Postmenopause.

## Biomarker differences

### Full dataset

**Fig. 1:**
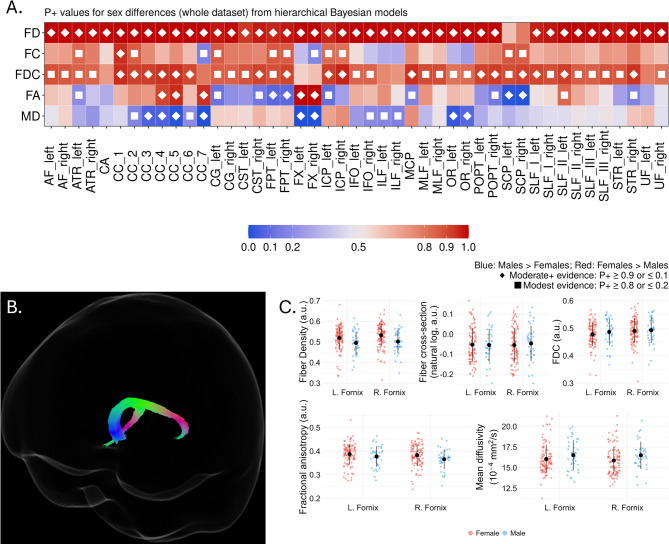
Sex differences in white matter metrics between men and women. (**A**) Heatmap showing tract-wise sex differences for FD, FC, FDC, FA, and MD across 45 bundles. Colors denote Female > Male (reds) or Male > Female (blues), with darker shades indicating stronger evidence. Diamonds indicate stronger evidence (*P* < 0.1, *P* > 0.9) and filled squares indicate more modest evidence (*P* < 0.2, *P* > 0.8). (**B**) Segmentation of the bilateral fornix. (**C**) Boxplots of residualized mean FD, FC, FDC, FA, and MD in the corpus callosum for females (red) and males (blue), adjusted for covariates. Black dots and lines indicate mean ± standard deviation. a.u.: Arbitrary units.

Figure 1A provides an overview of the direction and strength of evidence for sex differences across all tracts. Sex differences in white matter tract fiber properties were apparent throughout the brain and were not specific to any one fiber tract (see Fig. 1A). FD and FDC were greater throughout the majority of the brain in women, with no tracts identified with men having greater values. Women had evidence of greater FC in only 22.2% of the tracts, indicating that differences in FDC are driven by differences in FD, independent of FC as well as differences in total brain volume.

Additionally, men had greater FA in a total of 9 tracts (P + < 0.1: 4 tracts; Fig. 1A), which included the left anterior and right superior thalamic radiata, frontopontine tracts, and cerebellar peduncle tracts. Women had evidence of greater white matter greater FA, lower MD, or both compared to men in 12 tracts. These were distributed in the corpus callosum, bilateral fornix, bilateral superior and inferior longitudinal fasciculi, and optic radiata. By way of example, representative evidence in Fig. 1B shows the bilateral fornix and Fig. 1C presents data for each scalar type in the fornix residualized for APOE-ε4 carriership, family ADRD history, age, education, and total brain volume (for FC and FDC only). As evidenced by the moderate-to-strong evidence in the hierarchical Bayesian models (Fig. 1A), mean values for FD and FA were higher in the women and lower in the men, while men had greater average MD values in both fornices.

### Premenopause

**Fig. 2:**
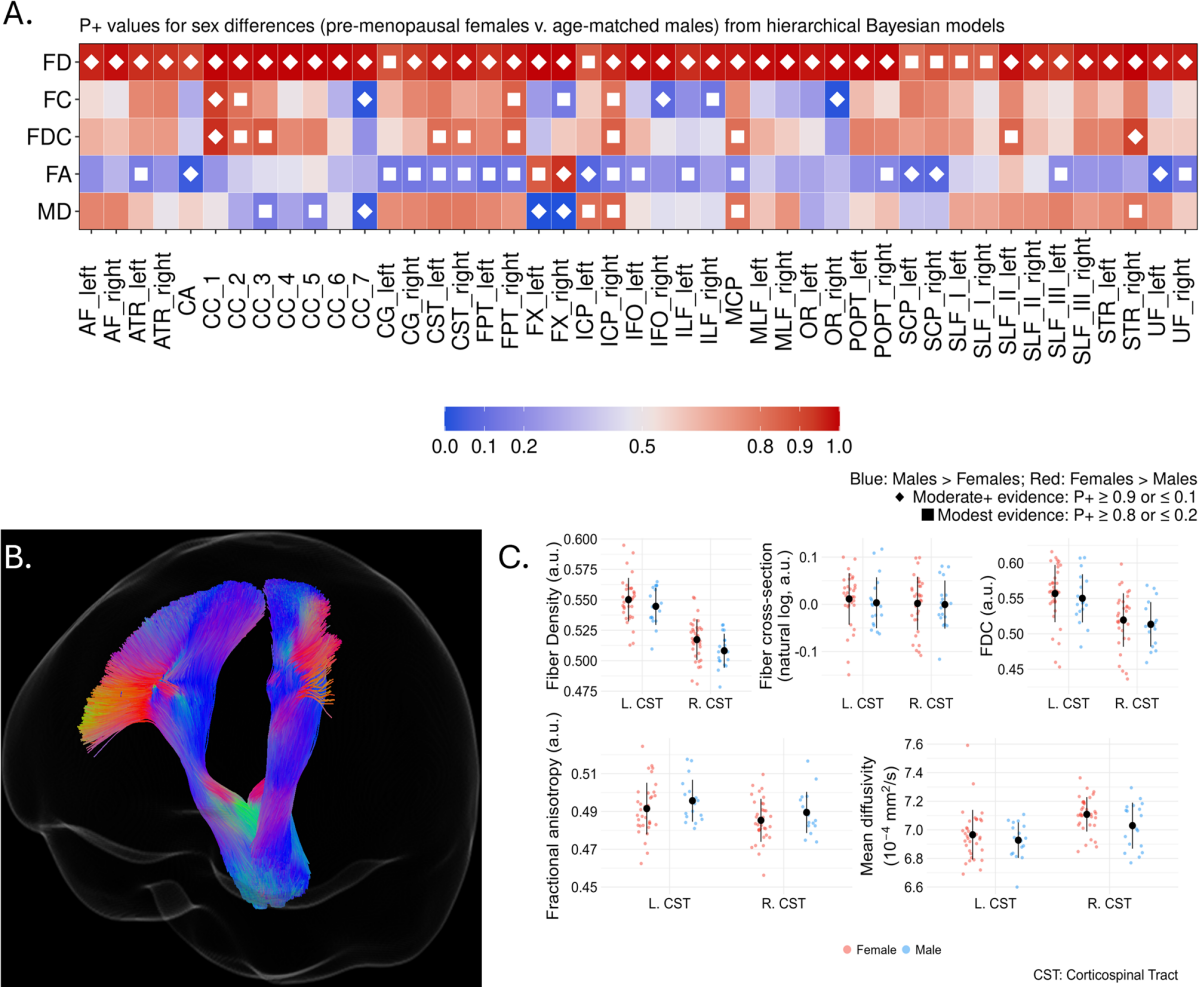
Sex differences in white matter metrics between premenopausal women and age-controlled men. (**A**) Heatmap showing tract-wise sex differences for FD, FC, FDC, FA, and MD across 45 bundles. Colors denote Female > Male (reds) or Male > Female (blues), with darker shades indicating stronger evidence. Diamonds indicate stronger evidence (*P* < 0.1, *P* > 0.9) and filled squares indicate more modest evidence (*P* < 0.2, *P* > 0.8). (**B**) Segmentation of the bilateral corticospinal tract. (**C**) Boxplots of residualized mean FD, FC, FDC, FA, and MD in the corpus callosum for females (red) and males (blue), adjusted for covariates. Black dots and lines indicate mean ± standard deviation. a.u.: Arbitrary units.

Figure 2A provides an overview of the directionality and strength of evidence for sex differences between the pre-menopausal women and age-controlled men. See Supplemental Tables 6–10 for full details including tract-wise mean values, model identified differences, 95% HDI, and P + values. In this group of women, we observed greater FD in all tracts. Greater FC and FDC were also observed in corpus callosum, frontopontine, cerebral peduncles. Age-controlled men exhibited evidence of greater FC in five tracts, including the splenium of the corpus callosum and the right fornix.

As shown in Fig. 2A, women exhibited greater FA in the bilateral fornix, as well as lower MD in segments of the corpus callosum. By contrast, men had greater FA in a wider range of tracts including left anterior thalamic radiation, cingulum bundle, corticospinal tracts, inferior and superior cerebellar peduncles, and uncinate fasciculi.

By way of example, representative evidence of these differences in the bilateral corticospinal tract is presented in Fig. 2B-C. After residualizing for other covariates, mean values for FD and FDC were greater in the women while men had greater FA.

### Perimenopause

**Fig. 3:**
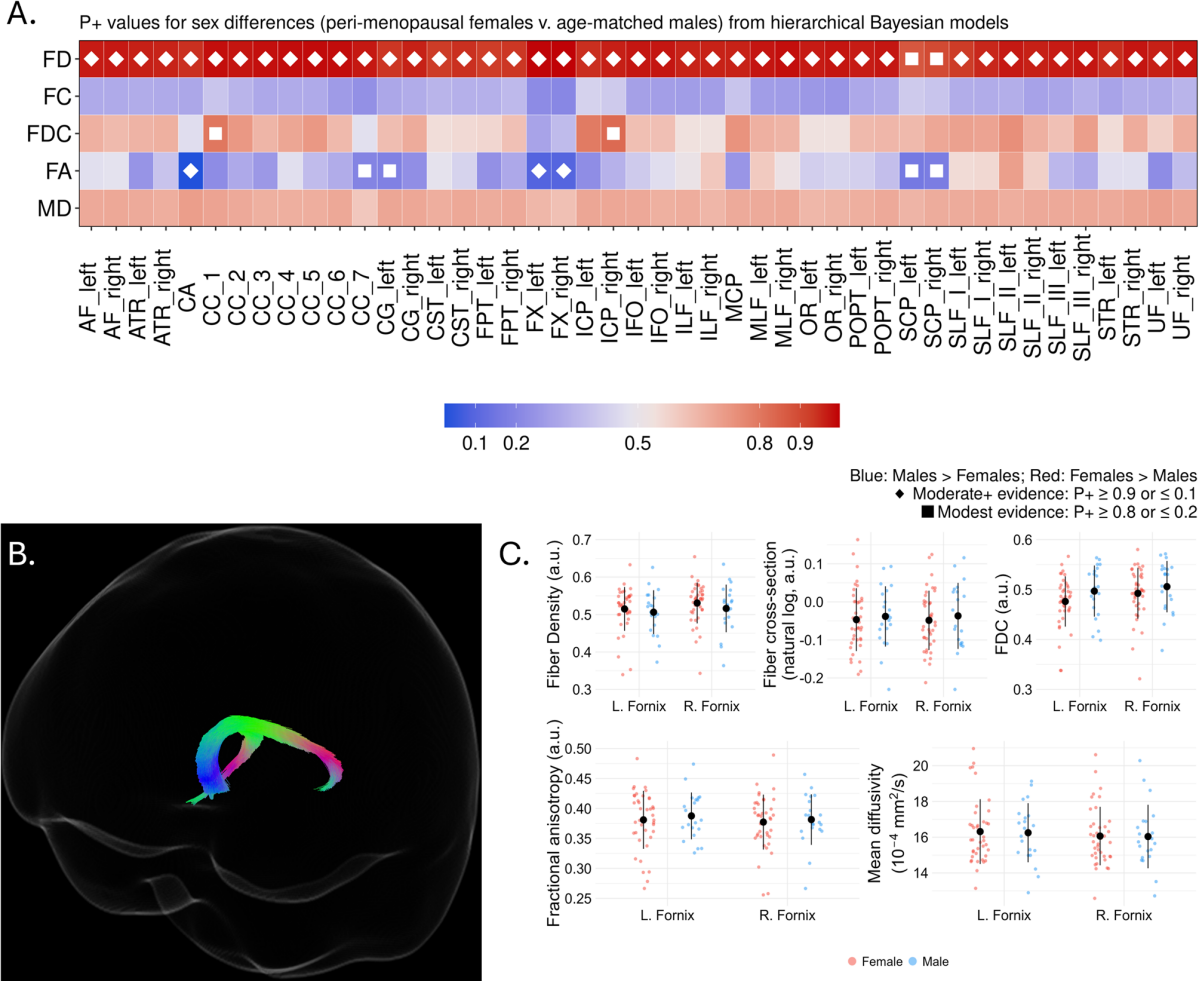
Sex differences in white matter metrics between perimenopausal women and age-controlled men. (**A**) Heatmap showing tract-wise sex differences for FD, FC, FDC, FA, and MD across 45 bundles. Colors denote Female > Male (reds) or Male > Female (blues), with darker shades indicating stronger evidence. Diamonds indicate stronger evidence (*P* < 0.1, *P* > 0.9) and filled squares indicate more modest evidence (*P* < 0.2, *P* > 0.8). (**B**) Segmentation of the bilateral fornix. (**C**) Boxplots of residualized mean FD, FC, FDC, FA, and MD in the corpus callosum for females (red) and males (blue), adjusted for covariates. Black dots and lines indicate mean ± standard deviation. a.u.: Arbitrary units.

Qualitatively, the white matter profile that emerged during the perimenopause was more consistent with a general absence of cross-metric sex differences that was distinct from both the pre- and the post-menopausal white matter profiles. There was strong evidence of greater FD in perimenopausal women compared to age-controlled men across all tracts (45/45) (Fig. 3A). There was additional evidence of greater FDC in the women in only two tracts. Greater FA was observed in the men in seven tracts, including the bilateral fornix, which qualitatively reverses the sex difference observed in pre- and post-menopausal profiles. There was no evidence of sex differences in FC or MD.

By way of example, Fig. 3B-C provides representative findings from the bilateral fornix. FD is greater in women while men had greater bilateral FA. Findings from the hierarchical models demonstrated that FC, FDC, and mean diffusivity were comparable between men and women. See Supplemental Tables 11–15 for full details including tract-wise mean values, model identified differences, 95% HDI, and P + values.

### Postmenopause

The white matter profile that emerged in the post-menopausal female brain exhibited the greatest extent of sex difference across white matter fiber micro- and macrostructure.

**Fig. 4:**
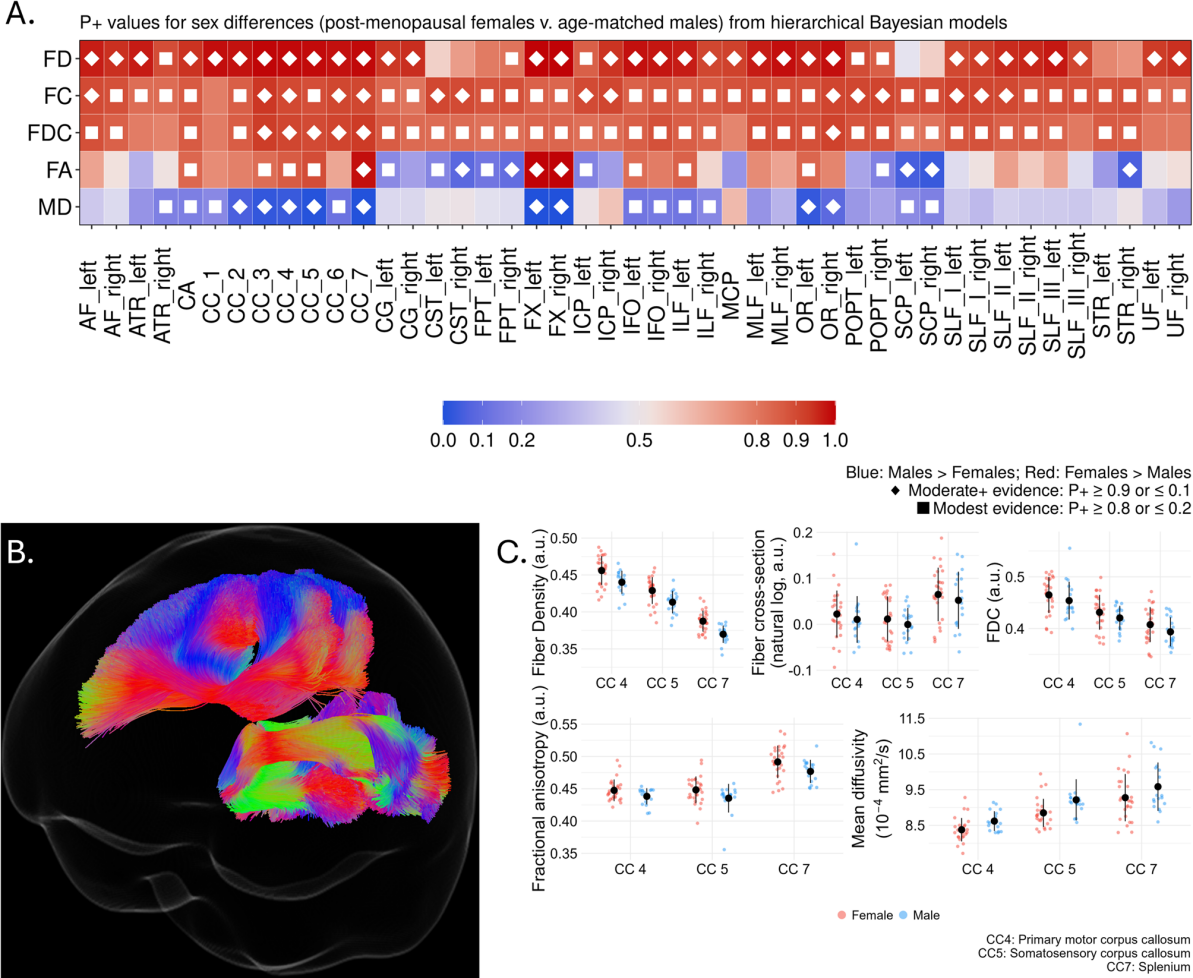
Sex differences in white matter metrics between postmenopausal women and age-controlled men. (**A**) Heatmap showing tract-wise sex differences for FD, FC, FDC, FA, and MD across 45 bundles. Colors denote Female > Male (reds) or Male > Female (blues), with darker shades indicating stronger evidence. Diamonds indicate stronger evidence (*P* < 0.1, *P* > 0.9) and filled squares indicate more modest evidence (*P* < 0.2, *P* > 0.8). (**B**) Segments 4, 5, and 7 of the corpus callosum. (**C**) Boxplots of residualized mean FD, FC, FDC, FA, and MD in the corpus callosum for females (red) and males (blue), adjusted for covariates. Black dots and lines indicate mean ± standard deviation. a.u.: Arbitrary units.

The post-menopausal women had evidence of greater FD, FC, or FDC in every tract compared to age-controlled men (Fig. 4A). Qualitatively, it is noteworthy for this group that whereas pre- and perimenopausal women had strong evidence greater FD in every nearly every tract, here there are 7 tracts without evidence of a difference and, while still the majority of the tracts, the number with moderate-to-strong evidence of a difference is down to 33/45.

As with findings from the whole dataset as well as the premenopausal age group comparison, postmenopausal women had greater FA in the bilateral fornix and splenium of the corpus callosum compared to men, while men exhibited greater FA in frontopontine tracts and cerebellar peduncle-related fiber bundles as well as the corticospinal tracts, left cingulum, and the right superior thalamic radiation. There was further evidence of greater MD in the men throughout commissural tracts (corpus callosum, anterior commissure) as well as the optic radiata, and bilateral inferior orbital and longitudinal tracts.

By way of example, Fig. 4B-C highlights representative findings from three segments of the corpus callosum: the primary motor segment, somatosensory segment, and the splenium. In all three segments, women had greater FD, FC, FDC, and FA while the men had greater mean diffusivity. See Supplemental Tables 15–20 for full details including tract-wise mean values, model identified differences, 95% HDI, and P + values.

## Discussion

Present findings indicate that, among midlife individuals with at least one genetic risk factor for AD, women have greater fiber density throughout the brain relative to men. These findings were consistent in pre- and perimenopausal groups, while the number of the tracts exhibiting moderate-to-strong evidence of greater values was decreased by 27% in the postmenopausal group. Evidence of wide-spread sex differences in diffusivity-based white matter FA and MD, which are indices of microstructural coherence as well as other tissue properties^41^, was sparse, which is broadly consistent with reported sex differences throughout the literature^2,12,16–21^, and modulated by menopausal transition status. Women on average, and specifically pre- and post-menopausal women, had greater FA and lower MD in the bilateral fornix as well as the corpus callosum compared to age-controlled men, whereas men had greater FA in the cerebellar peduncles, cingulum, and anterior commissure. Collectively, the findings indicate more globally tightly packed white matter along with greater white matter microstructural coherence in the fornix and corpus callosum in pre- and post-menopausal women. Results were independent of age, years of education, APOE genotype, and family history of AD, all of which may additionally and critically impact white matter health across midlife and later adulthood. The present findings extend a currently small literature base examining sex differences in fixel-based micro- and macrostructural outcomes^36–38^.

Choy et al. (2020) used fixel-based metrics to examine aging in 293 individuals aged 21–86 and found a general pattern of age-related decreases in FD, FC, and FDC^36^. Across these three metrics, sex differences were only observed in FD for the anterior thalamic radiation and the hippocampal portion of the cingulum; however the authors did not indicate which sex had the greater mean value for either of these bundles. Tinney et al. (2024) further examined age-related changes in FD, FC, and FDC in individuals aged 65–80. Men and women both exhibited decreasing FD, FC or FDC with age, and this effect was strongest in males, while women had smaller FC than men in subset of fixels exhibiting a positive age-FC relationship^38^. Those findings suggest a common age-related decrease in micro- and macrostructure with sex-specific trajectories for white matter changes in older adults^38^. By contrast, we observed consistent evidence supporting the interpretation that, at younger ages, women have greater FD throughout the brain and that there were no tracts in which men had greater density. We further observed sparse evidence for FC differences while FDC was greater in the women throughout the brain.

The observed differences between the present study and both Choy et al. (2020) and Tinney et al. (2024) may be reasonably explained by both the ages considered, as our sample did not include individuals younger than 36 or older than 65, as well as our examination of sex differences independent of age, rather than within the subset of areas demonstrating age effects. Further, both of the previous studies employed substantially different methodologies - including the use of multi-shell diffusion acquisitions and multi-shell multi-tissue CSD for creating fiber orientation distributions, the use of a volumetric atlas^36^ or fixelwise analyses^38^, the inclusion of total intracranial volume as a covariate in FD models (which is not recommended^42^, and the overall statistical frameworks - all of which impact cross-study comparability. Future work should thoroughly investigate the effects of these methodological choices on the ability to identify and understand underlying sex differences in white matter.

Interestingly, the greater fiber bundle packing in women did not manifest as extensively in the diffusion tensor metrics for these fiber bundles. Women across the menopausal transition stages had evidence of greater fractional anisotropy in only 6 bundles, including the fornix and segments of the corpus callosum. These findings agree with past work demonstrating greater FA in women in the fornix^12,13,16,17,19,43^ and the corpus callosum^12,17,23,44^. Men in this sample had greater mean diffusivity in these same tracts. This would be consistent with an interpretation that, for comparable fiber cross-sections, males’ less tightly packed bundles may allow for increased omnidirectional diffusivity. Men additionally had greater FA in tracts including the inferior, middle, and superior cerebellar peduncles, superior and anterior thalamic radiata, as well as corticospinal and the frontopontine tracts, consistent with previous work^10,16,17,23,44^. We expand on this below, but the tracts with cross-metric female advantage include limbic pathways, which are particularly sensitive to estrogenic effects^23,45,46^, suggesting that sex hormones may at least partially explain these differences.

Collectively, these findings suggest that, after accounting for age, total brain volume, and other confounders, men and women in this sample had generally comparable bundle cross-section, while women had more tightly packed white matter. This female advantage in fiber density may be reflected by greater FA and lower MD in women in tracts that are susceptible to both aging and neurodegenerative effects^9,12,34,35,47,48^. In light of the decreased FD observed in MCI and AD^34,35^ and the vulnerability of the female brain to AD, the findings in this cognitively normal, at-risk population may suggest either that white matter changes occur after the prodromal period or that these particular women are, on average, potentially resilient despite their risk factors. While either of these interpretations is plausible, both would require longitudinal data to substantiate them.

### Menopause as a modulator

Menopausal stage stratified analyses provided additional insights into the nature of the observed differences. The observed FD patterns were largely consistent across all three menopause stratified analyses. By contrast, sex differences in FC, FDC, FA, and mean diffusivity observed at both pre- and postmenopause were largely absent in perimenopause, including a complete reversal of the directionality of the difference in FA in the fornix. These findings point to a potential U-shaped distribution with advancing endocrinological age, whereby perimenopausal women exhibit similar microstructural characteristics to comparably aged men. Additionally, the strength of evidence for FD differences between men and women was diminished at postmenopause compared to premenopause, suggesting possibly permanent, and potentially destructive, remodeling of the white matter that occurs during perimenopause and those who are least able to recover those microstructural differences may be those at greatest risk for earlier neurodegenerative onset.

Several lines of work lend support for this interpretation. First, recent work has identified that circulating hormone levels, including 17β-estradiol and progesterone, are associated with dynamic changes in white matter diffusion and anisotropy properties across the menstrual cycle^45^, highlighting the temporal role of these hormones on white matter. This is further reflected by work suggesting that androgen and estrogen may confer masculinizing and feminizing effects, respectively, on white matter whereby the changing availability of and sensitivity to estrogen in the perimenopausal brain may briefly appear more like the male brain^23^. Second, estrogen receptors (ERs) are highly expressed in oligodendrocytes and astrocytes in white matter^49,50^ and are critical orchestrators of estrogen-mediated neuroprotective signaling pathways^51^, oxidative processes, and metabolism^52,53^. ER density increases during menopausal transition in estrogen-regulated networks^46^, in agreement with findings of higher density in ovariectomized rats^54^. Finally, menopause-specific work demonstrates that the postmenopausal female brain undergoes multiple adaptations in both gray and white matter structure and organization, bioenergetics, and cognition^2–4^. Collectively, these results align with the notion that women’s brains undergo a dynamic remodeling during the menopause transition, which in this study extends to white matter fiber bundles and some connectivity measures.

Sex differences in cerebral glucose metabolism have also been implicated in the sex-specific AD prevalence and differences in disease onset, pathophysiology and pathology progression as well as symptom presentation^2–6,55–59^. The declining regulatory effect of estrogen on glucose metabolism during menopause is thought to be a key driver of AD onset in women^50,60^. Animal studies demonstrate a catabolic effect on white matter in response to glucose hypometabolic states^61^, necessitating a remodeling of white matter pathways and a further bioenergetic adaptive response to preserve cognitive function^2^. Collectively, this body of work suggests that the bioenergetic adaptations required during menopause may, in part, be fulfilled through myelin catabolism until a more complete adaptive response can be mounted. The speed of this response dictates the extent to which white matter is remodeled and degraded, and as such may be a critical tipping point between neurodegenerative disease and resilience.

The current analysis excluded active MHT users and women who had undergone either a hysterectomy or oophorectomy, as these variables could confound detection of biomarker associations with menopause status. A natural extension of this work will be to explore interventions that could stabilize estrogen levels to support neuroendocrine aging in midlife women. Current guidelines recommend considering estrogen therapy, along with progestogen if the uterus is present, for cognitive support in women experiencing early menopause or premature ovarian insufficiency^62^. This is particularly the case following oophorectomy, where it is recommended to start hormone therapy as soon as possible after surgery and continue treatment until the average age of spontaneous menopause (around age 51), although the duration can vary based on individual factors and ongoing reassessment of benefits and risks. For women undergoing spontaneous menopause, as in this study, MHT is not currently recommended for AD risk reduction^62,63^. By identifying biological substrates of the menopause transition, present findings provide novel endpoints for clinical trials and observational research aimed at mitigating AD risk in menopausal women.

While our cohort was enriched with individuals at risk for AD (family history and/or APOE-ε4 carriership), current findings do not indicate that the women in our cohort have AD. Rather, present data provide insights into the possible intersection of menopause and AD risk on white matter micro- and macrostructure, supporting the notion that menopause-related hypoestrogenism may activate existing vulnerabilities to AD in some women^64^. Further research is warranted to simultaneously explore biomarkers of connectivity/diffusivity and AD pathology. We caution that the present results are cross-sectional and obtained from relatively small samples of carefully screened, highly educated participants at risk for AD. Replication in community-based populations with different risks and more diverse racial or socioeconomic backgrounds is warranted.

### Strengths and limitations

The present study has a number of strengths. First, we focused on carefully screened, healthy men and women ages 36–65 years, with comprehensive clinical and cognitive exams and menopause assessments. All of the individuals in this sample were cognitively normal at the time of participation, and our sample is enriched for AD risk factors, including APOE-ε4 carriership and family history. These factors increase the likelihood that the observed differences facilitate a midlife interpretation of potentially associated AD risk factors. Second, the fixel-based approach employed here reflects within-voxel fiber properties, distinguishing between microstructural fiber density contributions and fiber cross-section and bundle-level macrostructural morphological features. This interpretative framework is increasingly being applied, not just in methodological development^32^, but in applied research focused on human development, aging, neurodegeneration, and mental health^34,37,38,47,65–68^. The application to midlife sex differences is largely absent, and this work adds novel, targeted information in this area. Third, our hierarchical Bayesian approach enabled us to more completely characterize the probability of differences in these white matter metrics. This strategy provides prior information to be incorporated systematically into future studies examining white matter characteristics in aging and disease. Finally, our stratified analyses reflect the differences in endocrinological aging between men and women. There is no clearly defined, naturally occurring midlife analog in men for the endocrinological changes across the menopausal transition continuum, making chronological age the best proxy for this. By providing stratified analyses using age-controlled men for each menopausal transition stage, our analyses minimize comparing menopausally staged women to men at different proxy stages, improving endocrinologically informed interpretations.

Several limitations should be noted. First, the diffusion weighted imaging data collected were single-shell with a b-value of 1000s/mm^2^. Recommendations for fixel-based analyses include multi-shell data and higher b-values to better resolve crossing fibers^30^. Additionally, low b-values increase the extra-cellular contribution when computing FD^32^ and so we cannot rule out the possibility that the sex differences here reflect differences in both extra-cellular architecture and intra-axonal volume. However, past work, including some by the developers of the fixel-based metrics^32,65,69^, has successfully applied this to single-shell data, and we view the present findings as hypothesis-generating for future studies that can capitalize on multi-shell acquisitions and higher b-value data to refine the specificity of the findings here. Additionally, there are other contributors to white matter health that are not represented in these analyses, including lifestyle factors (e.g. smoking, alcohol, exercise), male-specific factors, overall health factors (e.g. diabetes, cardiovascular disease) as well as environmental considerations (e.g. socioeconomic status and childhood deprivation, pollution). Future work with larger samples should include these as potential factors that may alter the trajectory of white matter remodeling in midlife. Finally, these cross-sectional data were based on a relatively small sample size, particularly when stratified by menopause status, from a specific geographic region, which limits generalizability and does not allow for precise causal inference about the effect of endocrinological aging in women or male-specific midlife factors. Future studies should include larger samples and longitudinal designs to enable more precise modeling of the time course of these changes. Additional studies are needed in order to examine effects of menopause hormone therapy and as well as surgically induced menopause on the observed sex differences.

## Conclusions

Our findings demonstrate sex differences in white matter micro- and macrostructure across the menopausal transition, which were least pronounced at the perimenopausal stage and most pronounced at postmenopause. Perimenopause has been previously identified as a critical window of intervention for AD risk reduction and our findings extend this need to white matter health as well. Identifying and tracking changes in white matter during neuroendocrinological transitions could facilitate timely interventions that preserve neuronal integrity and mitigate future cognitive decline.

## Methods

### Participants

This is a cross-sectional study drawn from a larger cohort of cognitively normal men and women aged 35–65 years, with a family history of late-onset AD and/or carrying one or more APOE-ε4 alleles. Participants were recruited at the Weill Cornell Medicine (WCM) Alzheimer’s Prevention Program between 2018 and 2024 by self-referral, flyers, and word of mouth.

Our inclusion and exclusion criteria were previously described^2–4^. Briefly, all participants had Montreal Cognitive Assessment (MoCA) scores *≥* 26 and normal cognitive test performance by age and education^2–4^. Exclusion criteria included medical conditions that may affect brain structure or function (e.g., stroke, any neurodegenerative diseases, major psychiatric disorders, hydrocephalus, demyelinating disorders such as Multiple Sclerosis, intracranial mass, and infarcts on MRI), use of psychoactive medications, and contraindications to brain imaging. All received medical, neurological, laboratory, cognitive and MRI exams, including volumetric MRI and DTI, within 3 months of each other.

A family history of late-onset AD was elicited using standardized questionnaires^2–4^. APOE genotype was determined using standard qPCR procedures. Those carrying one or two APOE-ε4 alleles were grouped as APOE-ε4 carriers. The patients’ sex was determined by self-report. Our study protocol involves a ~ 1:3 enrollment ratio of men to women, with approximately equal representation of premenopausal, perimenopausal, and postmenopausal statuses among women. Determination of menopausal status was based on the Stages of Reproductive Aging Workshop (STRAW-10) criteria^70^ with hormone assessments as supportive criteria^5^. Participants were classified as premenopausal (regular cycler), perimenopausal (irregular cyclers with interval of amenorrhea *≥* 60 days or *≥* 2 skipped cycles) and postmenopausal (no cycle for *≥* 12 consecutive months)^5^. Information on hysterectomy/oophorectomy status and MHT usage was obtained through review of medical history. MHT users and women with a history of oophorectomy and/or hysterectomy were excluded.

*Standard protocol approvals*,* registrations*,* and patient consents*. All methods were carried out in accordance with relevant guidelines and regulations. All experimental protocols were approved by the WMC Institutional Review Boards. Written informed consent was obtained from all participants.

### Image acquisition

All participants underwent MRI neuroimaging on a 3.0 Tesla G.E. Discovery MR750 scanner equipped with a 32-channel coil. Acquired sequences included a T1-weighted (T1w) 3D sagittal brain volume imaging (BRAVO) sequence acquired with isotropic 1 × 1 × 1 mm resolution (TR: 8.2ms; TE: 3.2ms; TI: 450ms; Flip angle: 12°; FOV: 25.6 cm on a 256 × 256 matrix; ARC acceleration). Diffusion Weighted Imaging (DWI) was acquired with one b = 0 s/mm^2^ volume and 55 b = 1000 s/mm^2^ directions (TR: 8 s; TE: 65ms; acquisition matrix: 128 × 128 reconstructed to 256 × 256; resolution: 0.9 × 0.9 × 1.8 mm).

### Image minimal pre-processing

#### CAT12

To estimate total brain volume (TBV), all T1-weighted images were processed using CAT12 (v. 12.8.2) in SPM12 using default parameters^71^. Outputs from the process included spatially normalized gray matter, white matter, and CSF maps as well as tissue-type specific volume measures and total brain volume.

#### SynB0-DISCO

Prior to pre-processing the diffusion weighted images, an undistorted, diffusion image was synthesized from the b = 0 diffusion image and the T1-weighted image using SyNB0-DISCO^72^. The purpose of this step was to enable susceptibility distortion correction.

#### QSIPrep

Pre-processed diffusion weighted data were produced using QSIPrep (v. 0.19.1)^73^, which is based on Nipype 1.8.6 (RRID: SCR_002502)^74,75^. Descriptions of the preprocessing steps are provided by boilerplates distributed with QSIPrep’s outputs under a CC0 license for the purpose of transparency and reproducibility in published works. These are presented below with minimal modifications for formatting and clarity.

**Anatomical preprocessing.** The T1-weighted image was corrected for intensity non-uniformity (INU) using N4BiasFieldCorrection (ANTs 2.4.3)^76^, and used as an anatomical reference throughout the workflow. The anatomical reference image was reoriented into AC-PC alignment via a 6-DOF transform extracted from a full affine registration to the MNI152NLin2009cAsym template. A full nonlinear registration to the template from AC-PC space was estimated via symmetric nonlinear registration (SyN) using *antsRegistration*. Brain extraction was performed on the T1w image using SynthStrip^77^ and automated segmentation was performed using SynthSeg^78,79^ from FreeSurfer version 7.3.1.

**Diffusion preprocessing.** MP-PCA denoising as implemented in MRtrix3’s *dwidenoise*^80,81^ was applied with a 5-voxel window. After MP-PCA, Gibbs unringing was performed using MRtrix3’s *mrdegibbs*^82^. Following unringing, the mean intensity of the DWI series was adjusted so all the mean intensity of the b = 0 images matched across each separate DWI scanning sequence. B1 field inhomogeneity was corrected using *dwibiascorrect* from MRtrix3 with the N4 algorithm^76^ after corrected images were resampled.

FSL (version 6.0.5.1:57b01774)’s *eddy* was used for head motion correction and Eddy current correction^83^. Eddy was configured with a q-space smoothing factor of 10, a total of 5 iterations, and 1000 voxels used to estimate hyperparameters. A linear first level model and a linear second level model were used to characterize Eddy current-related spatial distortion. q-space coordinates were forcefully assigned to shells. Field offset was attempted to be separated from subject movement. Shells were aligned post-eddy. Eddy’s outlier replacement was run^84^. Data were grouped by slice, only including values from slices determined to contain at least 250 intracerebral voxels. Groups deviating by more than 4 standard deviations from the prediction had their data replaced with imputed values. Data was provided with the synthesized undistorted image from SynB0-DISCO. Here, the synthesized undistorted image was used along with the b = 0 image extracted from the DWI scan. From this pair of images the susceptibility-induced off-resonance field was estimated using a method similar to that described in (Andersson, Skare, and Ashburner 2003)^85^. The fieldmaps were ultimately incorporated into the Eddy current and head motion correction interpolation. Final interpolation was performed using the *jac* method.

Several confounding time-series were calculated based on the preprocessed DWI: framewise displacement using the implementation in Nipype (following the definitions by Power et al. 2014)^86^. The head-motion estimates calculated in the correction step were also placed within the corresponding confounds file. Slicewise cross correlation was also calculated. The DWI time-series were resampled to ACPC, generating a preprocessed DWI run in ACPC space with 1 mm isotropic voxels.

Many internal operations of QSIPrep use Nilearn 0.10.2 (RRID: SCR_001362)^87,88^ and DIPY^89^. For more details of the pipeline, see the section corresponding to workflows in QSIPrep’s documentation (https://qsiprep.readthedocs.io*).*

### Fixel-based analyses

The fixel-based analysis pipeline was implemented in MRtrix3 (v. 3.0.4)^90^, has been described in detail in other publications^30,32^, and the present processing approach was adapted from a previous publication^67^. Briefly, all QSIPrep preprocessed T1w and DWI volumes and vector files as well as the brain mask were reoriented to FSL standard orientation. The preprocessed DWI volumes were denoised using DIPY’s *Patch2Self* algorithm to minimize residual noise and improve SNR^91^. Response functions for grey matter, white matter, and CSF were estimated in MRtrix3 using the *dhollander* algorithm^92,93^ and an average response function for each tissue type was created across the whole dataset^31^. Fiber orientation directions (FODs) for each participant were generated using the *MRtrix3Tissue* Single-Shell 3-Tissue Constrained Spherical Deconvolution method^69^ (v. 5.2.9; https://github.com/3Tissue/MRtrix3Tissue*)* and log-domain intensity normalized^30,32^.

A randomly selected sample of 10 women in each menopausal transition state (pre-, peri-, post-) as well as 10 age-range matched men for each transition state were used to generate an unbiased, study-specific FOD template. All participant FODs were then independently registered and warped to the template brain along with their brain masks. A whole-brain fixel-wise analysis mask was created from the FOD template within the intersection of the warped brain masks using MRtrix’s fod2fixel using a peak FOD amplitude minimum threshold of 0.09 for inclusion. Participants’ warped FODs were then segmented, reoriented, and mapped to template space. For each participant, fiber density (FD), fiber cross-section (FC), and the product of FD and FC (FDC) were calculated for each fixel. FC was additionally log-transformed prior to analyses due to a known non-normal distribution. All subsequent references to FC refer to the log-transformed values.

### Tract segmentation

The three primary spherical harmonic peaks from the FOD template brain were passed to TractSeg (v. 2.3)^94–97^. Tractograms were generated for 45 commissural and bilateral fiber bundles available in TractSeg. For each fiber bundle, the tractograms were converted to fixel masks using MRtrix’s tck2fixel. Finally mean values for FD, FC, and FDC were calculated for each participant for each fiber bundle.

### DTI scalar preparation

From the reoriented DWI volumes, two diffusion tensor scalars were computed (FA, MD) using an iteratively weighted least squares approach^90,98^. These were computed in the participant’s native space, warped to the FOD template space, and then mapped to the TractSeg fiber bundle using TractSeg’s *Tractometry* function^97^. The *Tractometry* function divides each fiber bundle into 100 equally sized segments and computes the mean scalar value for each segment. For the present analyses, the mean value for the middle 98 segments (discarding the first and last segments) was computed as the summary statistic, as recommended by the TractSeg developers, for each fiber bundle.

### Statistical analyses

All preprocessing and analyses were conducted on the high performance computing cluster at the University of Arizona. Analyses were conducted using R (v. 4.4.0)^99^ and the *groundhog* package (v. 3.1.2)^100^ was used for package version control, loading packages from 2024-05-01. The primary aim was to identify sex-specific differences in fiber bundles’ micro- and macrostructural properties. We employed hierarchical Bayesian regression models to assess these differences at the level of both the whole brain and individual fiber bundles simultaneously. Five models were fit (FDC, FC, FD, FA, and MD) using *brms*^101,102^, implementing 8 parallel chains and 10,000 iterations, plus an initial 5,000 tuning samples which were discarded, resulting in 80,000 total samples per model. The primary outcome of interest was the per-bundle differences between males and females. Each model incorporated previously identified predictors of white matter health, including age, years of education, APOE-ε4 carriership status (carrier vs. non-carrier), and family history of AD (positive vs. negative). Age, years of education, and total intracranial volume (FDC and FC models only) were Z-score normalized before modeling.

Bundle-specific differences between sexes were identified from the posterior distributions of each model. These outcomes are summarized across the entire posterior distribution, presenting sex-specific mean values adjusted for all other covariates, along with the 95% highest density interval (HDI) of the between-sex difference and the proportion of differences greater than zero (P+). P + provides a continuum of evidence, indicating the probability that the difference is in the observed direction^39,40^. As opposed to *p*-values, P + is not interpreted through dichotomization (e.g., significant vs. not significant). Instead P + in the present study indicates the probability of a female > male difference given the collected data. For example, P + = 0.9 for FA in a tract indicates a 90% probability of female FA > male FA while P + = 0.1 would indicate only a 10% probability, which implies that the probability of male FA > female FA is 90%. In line with the intention to provide a comprehensive view of sex differences in our data, descriptive statistics of all posterior distributions are provided in Supplementary Materials while the main reporting focuses on differences identified at both modest (P + > 0.8, P + < 0.2) and stronger (P + > 0.9, P + < 0.1) levels of evidence^103–105^.

Stratified analyses were then conducted to determine whether the observed sex differences could be attributed to menopausal status (pre-, peri-, and post-menopausal) or if they remained stable across the age range in our data, adjusting by the same confounders as above. To this end, the dataset was subdivided into three subsets, each containing women in a specific menopausal transition state and men matched to the same age range as the women in each of the menopausal status groups, consistent with prior research^2,6,106^. Due to overlapping age ranges for these transition state groups, a total of 20 men were included in more than one of the secondary analysis datasets; however these datasets were analyzed separately and are thus treated as independent. The same modeling approach was applied to each subset, and results were reported similarly.

## Supplementary Information

Below is the link to the electronic supplementary material.

Supplementary Material 1

## Data Availability

The datasets analyzed during the current study may be made available from the corresponding author on reasonable request.
