# Supplementary material for: White matter microstructural and macrostructural profiles during midlife reveal sex differences between men and women at different menopausal stages
Source: Sci Rep. 2025 Nov 17;15:40312. doi: 10.1038/s41598-025-24136-y (PMC12623426; doi:10.1038/s41598-025-24136-y)
Supplement: Supplementary file 1 — Supplementary Material 1 [file 41598_2025_24136_MOESM1_ESM.pdf]

Supplemental table 1: Fiber density differences between women and men

	Raw means[1]		Covariate adjusted means[1]		Hierarchical Bayesian model[1]		
Tract	Females	Males	Females	Males	Mean Difference	95% HDI	P+
AF_left	0.369±0.015	0.358±0.014	0.368±0.014	0.360±0.013	0.010	[0.004, 0.015]	0.998
AF_right	0.393±0.018	0.382±0.015	0.393±0.018	0.383±0.015	0.010	[0.004, 0.015]	0.998
ATR_left	0.367±0.017	0.359±0.018	0.366±0.017	0.361±0.015	0.009	[0.003, 0.014]	0.993
ATR_right	0.366±0.016	0.359±0.016	0.365±0.015	0.360±0.015	0.008	[0.002, 0.013]	0.987
CA	0.442±0.027	0.434±0.025	0.442±0.026	0.435±0.024	0.009	[0.003, 0.014]	0.994
CC_1	0.454±0.020	0.435±0.018	0.453±0.019	0.438±0.017	0.014	[0.009, 0.020]	1.000
CC_2	0.384±0.016	0.371±0.014	0.384±0.015	0.373±0.012	0.011	[0.006, 0.017]	0.999
CC_3	0.402±0.022	0.384±0.021	0.401±0.022	0.386±0.019	0.014	[0.008, 0.020]	1.000
CC_4	0.457±0.023	0.437±0.018	0.456±0.023	0.440±0.017	0.014	[0.009, 0.020]	1.000
CC_5	0.433±0.021	0.412±0.018	0.432±0.021	0.414±0.016	0.015	[0.009, 0.021]	1.000
CC_6	0.410±0.018	0.392±0.013	0.409±0.017	0.393±0.013	0.013	[0.008, 0.019]	1.000
CC_7	0.387±0.017	0.372±0.013	0.387±0.017	0.373±0.012	0.012	[0.007, 0.018]	1.000
CG_left	0.352±0.014	0.345±0.014	0.352±0.014	0.346±0.013	0.007	[0.002, 0.013]	0.979
CG_right	0.365±0.017	0.355±0.013	0.364±0.017	0.357±0.012	0.009	[0.003, 0.014]	0.994
CST_left	0.553±0.020	0.549±0.019	0.553±0.020	0.550±0.019	0.006	[0.000, 0.011]	0.939
CST_right	0.519±0.019	0.512±0.017	0.519±0.019	0.513±0.017	0.007	[0.001, 0.012]	0.973
FPT_left	0.477±0.017	0.472±0.016	0.476±0.017	0.473±0.016	0.006	[0.001, 0.012]	0.959
FPT_right	0.467±0.017	0.460±0.018	0.467±0.017	0.461±0.018	0.007	[0.001, 0.012]	0.975
FX_left	0.523±0.059	0.486±0.068	0.520±0.056	0.495±0.056	0.027	[0.021, 0.033]	1.000
FX_right	0.536±0.061	0.492±0.070	0.532±0.056	0.502±0.057	0.033	[0.027, 0.039]	1.000
ICP_left	0.416±0.018	0.411±0.018	0.416±0.018	0.412±0.017	0.006	[0.001, 0.012]	0.961
ICP_right	0.381±0.018	0.373±0.015	0.381±0.018	0.374±0.014	0.008	[0.003, 0.014]	0.990
IFO_left	0.389±0.015	0.375±0.016	0.388±0.015	0.377±0.013	0.012	[0.006, 0.017]	1.000
IFO_right	0.386±0.016	0.372±0.012	0.385±0.016	0.373±0.011	0.011	[0.006, 0.017]	1.000
ILF_left	0.389±0.017	0.378±0.016	0.389±0.016	0.380±0.015	0.010	[0.004, 0.015]	0.998
ILF_right	0.393±0.018	0.385±0.018	0.393±0.018	0.386±0.017	0.008	[0.003, 0.014]	0.990
MCP	0.456±0.019	0.445±0.016	0.455±0.019	0.447±0.014	0.009	[0.004, 0.015]	0.995
MLF_left	0.377±0.016	0.362±0.013	0.377±0.016	0.364±0.012	0.011	[0.006, 0.017]	0.999
MLF_right	0.382±0.018	0.364±0.014	0.381±0.018	0.366±0.013	0.013	[0.007, 0.019]	1.000
OR_left	0.395±0.016	0.383±0.015	0.394±0.016	0.384±0.013	0.010	[0.004, 0.015]	0.997
OR_right	0.404±0.018	0.389±0.014	0.404±0.018	0.391±0.013	0.011	[0.006, 0.017]	0.999
POPT_left	0.464±0.017	0.452±0.014	0.464±0.017	0.453±0.014	0.010	[0.004, 0.015]	0.997
POPT_right	0.463±0.017	0.450±0.014	0.462±0.017	0.451±0.014	0.010	[0.005, 0.016]	0.998
SCP_left	0.464±0.017	0.468±0.015	0.464±0.016	0.467±0.015	0.001	[-0.005, 0.007]	0.622
SCP_right	0.440±0.015	0.440±0.015	0.440±0.015	0.440±0.015	0.003	[-0.003, 0.008]	0.790
SLF_III_left	0.370±0.017	0.360±0.017	0.369±0.016	0.362±0.015	0.010	[0.004, 0.015]	0.997
SLF_III_right	0.378±0.020	0.367±0.017	0.377±0.019	0.369±0.017	0.009	[0.004, 0.015]	0.997
SLF_II_left	0.338±0.017	0.325±0.016	0.337±0.017	0.326±0.016	0.011	[0.005, 0.016]	0.999
SLF_II_right	0.332±0.018	0.322±0.014	0.332±0.017	0.323±0.014	0.009	[0.004, 0.015]	0.996
SLF_I_left	0.310±0.015	0.305±0.013	0.310±0.015	0.306±0.013	0.006	[0.001, 0.012]	0.965
SLF_I_right	0.329±0.017	0.323±0.011	0.329±0.017	0.324±0.010	0.007	[0.002, 0.013]	0.979
STR_left	0.495±0.022	0.488±0.019	0.495±0.022	0.488±0.019	0.007	[0.001, 0.013]	0.978
STR_right	0.471±0.021	0.457±0.019	0.471±0.021	0.458±0.019	0.011	[0.005, 0.016]	0.999
UF_left	0.426±0.018	0.416±0.019	0.426±0.018	0.417±0.018	0.010	[0.004, 0.015]	0.998
UF_right	0.416±0.019	0.405±0.017	0.416±0.019	0.406±0.016	0.010	[0.005, 0.016]	0.999

[1] Units for raw and adjusted means, difference, and 95% HDI are arbitrary.

Data are presented as mean ± SD. Raw values are unadjusted sex-wise means. Adjusted values are sex-wise mean residual values after adjusting for age, education, APOE e4 carriership, and family history of Alzheimer's disease. Bayesian model outputs include the mean difference and 95% highest density interval estimates as well as P+. P+ indicates the proportion of the Bayesian samples (N = 80000) where the difference between women and men was > 0. P+ values approaching 1 indicate stronger evidence of female > male while P+ values approaching 0 indicate stronger evidence of male > female. The hierarchical model includes all tracts in a singular model and leverages partial pooling of the data, therefore the mean difference will not always be exactly equal to the difference in covariate adjusted means.

Supplemental table 2: Fiber cross-section differences between women and men

Tract	Raw means [1]		Covariate adjusted means [1]		Hierarchical Bayesian model [1]		
	Females	Males	Females	Males	Mean Difference	95% HDI	P+
AF_left	0.008±0.056	0.088±0.073	0.031±0.035	0.025±0.039	0.004	[-0.009, 0.017]	0.695
AF_right	0.006±0.055	0.089±0.073	0.029±0.032	0.026±0.042	0.004	[-0.009, 0.017]	0.708
ATR_left	-0.017±0.070	0.069±0.082	0.009±0.047	-0.001±0.043	0.008	[-0.005, 0.022]	0.833
ATR_right	-0.013±0.070	0.073±0.083	0.012±0.048	0.004±0.053	0.007	[-0.007, 0.020]	0.784
CA	-0.011±0.062	0.068±0.083	0.009±0.051	0.014±0.063	0.001	[-0.012, 0.014]	0.557
CC_1	-0.022±0.073	0.044±0.084	0.000±0.053	-0.015±0.061	0.014	[0.000, 0.029]	0.945
CC_2	-0.003±0.066	0.074±0.081	0.021±0.043	0.008±0.047	0.010	[-0.004, 0.023]	0.867
CC_3	0.010±0.079	0.087±0.101	0.035±0.065	0.020±0.067	0.006	[-0.008, 0.020]	0.757
CC_4	-0.012±0.063	0.059±0.081	0.007±0.055	0.006±0.058	0.003	[-0.010, 0.016]	0.641
CC_5	-0.019±0.059	0.051±0.066	0.000±0.046	-0.002±0.044	0.005	[-0.008, 0.018]	0.745
CC_6	-0.026±0.052	0.057±0.070	-0.003±0.033	-0.003±0.048	0.003	[-0.010, 0.015]	0.627
CC_7	0.017±0.070	0.129±0.092	0.043±0.058	0.058±0.070	-0.009	[-0.024, 0.006]	0.162
CG_left	-0.031±0.059	0.048±0.071	-0.008±0.036	-0.013±0.044	0.009	[-0.004, 0.023]	0.865
CG_right	-0.027±0.056	0.051±0.066	-0.005±0.034	-0.007±0.043	0.007	[-0.007, 0.020]	0.791
CST_left	-0.015±0.061	0.036±0.064	0.000±0.054	-0.005±0.048	0.006	[-0.007, 0.019]	0.781
CST_right	-0.021±0.059	0.036±0.071	-0.004±0.051	-0.010±0.052	0.006	[-0.007, 0.019]	0.763
FPT_left	-0.016±0.052	0.046±0.067	0.003±0.037	-0.004±0.041	0.009	[-0.004, 0.023]	0.870
FPT_right	-0.011±0.055	0.047±0.069	0.008±0.041	-0.004±0.043	0.009	[-0.004, 0.022]	0.863
FX_left	-0.092±0.107	0.050±0.120	-0.053±0.083	-0.055±0.082	-0.004	[-0.019, 0.012]	0.353
FX_right	-0.092±0.104	0.055±0.124	-0.055±0.084	-0.046±0.088	-0.009	[-0.026, 0.007]	0.179
ICP_left	-0.027±0.064	0.018±0.055	-0.014±0.056	-0.017±0.050	0.008	[-0.005, 0.022]	0.830
ICP_right	-0.026±0.061	0.017±0.056	-0.013±0.053	-0.019±0.049	0.008	[-0.006, 0.021]	0.818
IFO_left	0.005±0.057	0.097±0.071	0.029±0.037	0.032±0.036	0.001	[-0.012, 0.014]	0.543
IFO_right	0.005±0.055	0.103±0.073	0.029±0.038	0.038±0.050	-0.003	[-0.016, 0.011]	0.372
ILF_left	0.023±0.057	0.120±0.072	0.048±0.038	0.054±0.040	-0.004	[-0.019, 0.009]	0.307
ILF_right	0.016±0.061	0.121±0.077	0.042±0.042	0.049±0.046	-0.005	[-0.018, 0.009]	0.302
MCP	-0.012±0.063	0.038±0.061	0.002±0.055	-0.001±0.052	0.005	[-0.008, 0.019]	0.746
MLF_left	-0.007±0.052	0.077±0.066	0.015±0.031	0.016±0.041	0.001	[-0.012, 0.014]	0.560
MLF_right	-0.017±0.052	0.069±0.070	0.006±0.034	0.009±0.045	0.002	[-0.010, 0.016]	0.622
OR_left	0.013±0.066	0.110±0.086	0.038±0.052	0.043±0.053	-0.004	[-0.018, 0.010]	0.326
OR_right	-0.004±0.066	0.102±0.087	0.021±0.054	0.034±0.069	-0.006	[-0.020, 0.008]	0.254
POPT_left	-0.025±0.051	0.038±0.057	-0.006±0.037	-0.011±0.037	0.005	[-0.008, 0.018]	0.752
POPT_right	-0.028±0.050	0.033±0.060	-0.011±0.037	-0.015±0.042	0.006	[-0.007, 0.019]	0.778
SCP_left	-0.030±0.049	0.009±0.057	-0.017±0.041	-0.026±0.044	0.010	[-0.004, 0.024]	0.880
SCP_right	-0.032±0.048	0.009±0.055	-0.019±0.041	-0.026±0.043	0.009	[-0.005, 0.022]	0.860
SLF_III_left	0.005±0.061	0.091±0.086	0.029±0.043	0.026±0.059	0.003	[-0.010, 0.016]	0.624
SLF_III_right	0.009±0.060	0.084±0.076	0.030±0.041	0.026±0.057	0.005	[-0.008, 0.018]	0.727
SLF_II_left	0.017±0.061	0.090±0.073	0.039±0.041	0.029±0.042	0.005	[-0.009, 0.018]	0.708
SLF_II_right	0.010±0.065	0.095±0.075	0.034±0.044	0.030±0.045	0.004	[-0.010, 0.017]	0.670
SLF_I_left	0.009±0.053	0.072±0.066	0.028±0.040	0.023±0.044	0.003	[-0.010, 0.017]	0.655
SLF_I_right	0.000±0.056	0.073±0.064	0.021±0.039	0.018±0.042	0.004	[-0.009, 0.017]	0.693
STR_left	-0.034±0.061	0.033±0.070	-0.017±0.054	-0.011±0.056	0.005	[-0.009, 0.018]	0.714
STR_right	-0.019±0.057	0.034±0.073	-0.004±0.050	-0.007±0.053	0.007	[-0.006, 0.020]	0.799
UF_left	-0.011±0.056	0.074±0.062	0.011±0.038	0.015±0.031	0.004	[-0.010, 0.017]	0.669
UF_right	0.002±0.053	0.083±0.066	0.023±0.036	0.026±0.041	0.002	[-0.011, 0.015]	0.617

[1] Units for raw and adjusted means, difference, and 95% HDI are arbitrary.

Data are presented as mean ± SD. Raw values are unadjusted sex-wise means. Adjusted values are sex-wise mean residual values after adjusting for age, education, intracranial volume, APOE e4 carriership, and family history of Alzheimer's disease. Bayesian model outputs include the mean difference and 95% highest density interval estimates as well as P+. P+ values approaching 1 indicate stronger evidence of female > male while P+ values approaching 0 indicate stronger evidence of male > female. The hierarchical model includes all tracts in a singular model and leverages partial pooling of the data, therefore the mean difference will not always be exactly equal to the difference in covariate adjusted means.

Supplemental table 3: FDC differences between women and men

	Raw means[1]		Covariate adjusted means[1]		Hierarchical Bayesian model[1]		
Tract	Females	Males	Females	Males	Mean Difference	95% HDI	P+
AF_left	0.375±0.029	0.394±0.035	0.382±0.024	0.375±0.024	0.007	[-0.002, 0.017]	0.888
AF_right	0.397±0.029	0.419±0.036	0.405±0.025	0.400±0.027	0.007	[-0.003, 0.017]	0.873
ATR_left	0.362±0.029	0.385±0.035	0.369±0.023	0.364±0.022	0.006	[-0.003, 0.017]	0.852
ATR_right	0.362±0.028	0.386±0.035	0.369±0.023	0.366±0.028	0.006	[-0.004, 0.016]	0.845
CA	0.438±0.042	0.466±0.053	0.445±0.039	0.446±0.045	0.004	[-0.007, 0.014]	0.712
CC_1	0.447±0.039	0.453±0.048	0.453±0.035	0.440±0.040	0.011	[0.000, 0.021]	0.952
CC_2	0.385±0.031	0.399±0.036	0.391±0.026	0.383±0.027	0.009	[-0.001, 0.018]	0.920
CC_3	0.407±0.043	0.418±0.044	0.414±0.039	0.401±0.036	0.009	[-0.001, 0.019]	0.928
CC_4	0.455±0.043	0.466±0.042	0.459±0.042	0.454±0.036	0.010	[0.000, 0.020]	0.941
CC_5	0.427±0.038	0.434±0.035	0.430±0.037	0.424±0.029	0.010	[0.000, 0.020]	0.946
CC_6	0.402±0.031	0.417±0.034	0.407±0.028	0.402±0.027	0.008	[-0.002, 0.018]	0.906
CC_7	0.393±0.037	0.420±0.042	0.400±0.034	0.400±0.033	0.005	[-0.005, 0.015]	0.799
CG_left	0.343±0.025	0.364±0.032	0.349±0.021	0.348±0.027	0.007	[-0.003, 0.017]	0.859
CG_right	0.357±0.026	0.376±0.030	0.362±0.022	0.361±0.024	0.006	[-0.003, 0.016]	0.852
CST_left	0.547±0.044	0.568±0.040	0.554±0.042	0.549±0.033	0.007	[-0.003, 0.018]	0.882
CST_right	0.512±0.039	0.530±0.039	0.518±0.037	0.513±0.034	0.009	[-0.001, 0.019]	0.918
FPT_left	0.471±0.034	0.493±0.039	0.478±0.030	0.473±0.029	0.007	[-0.002, 0.017]	0.888
FPT_right	0.463±0.033	0.481±0.040	0.470±0.029	0.462±0.032	0.008	[-0.002, 0.018]	0.913
FX_left	0.472±0.044	0.501±0.057	0.478±0.042	0.487±0.049	0.001	[-0.010, 0.012]	0.575
FX_right	0.485±0.048	0.507±0.058	0.490±0.044	0.493±0.047	0.002	[-0.009, 0.013]	0.617
ICP_left	0.408±0.034	0.421±0.026	0.412±0.032	0.411±0.025	0.009	[-0.001, 0.019]	0.931
ICP_right	0.373±0.030	0.380±0.024	0.377±0.028	0.370±0.021	0.011	[0.001, 0.021]	0.959
IFO_left	0.390±0.028	0.410±0.032	0.396±0.024	0.392±0.020	0.006	[-0.004, 0.016]	0.844
IFO_right	0.385±0.028	0.407±0.031	0.392±0.025	0.390±0.022	0.006	[-0.004, 0.016]	0.848
ILF_left	0.398±0.030	0.424±0.036	0.405±0.026	0.405±0.025	0.004	[-0.006, 0.014]	0.756
ILF_right	0.398±0.035	0.431±0.040	0.407±0.029	0.405±0.028	0.004	[-0.006, 0.014]	0.731
MCP	0.455±0.040	0.468±0.034	0.460±0.038	0.454±0.030	0.010	[0.000, 0.020]	0.945
MLF_left	0.377±0.028	0.392±0.029	0.382±0.024	0.377±0.022	0.008	[-0.002, 0.018]	0.899
MLF_right	0.377±0.030	0.392±0.032	0.383±0.027	0.378±0.024	0.008	[-0.002, 0.018]	0.895
OR_left	0.397±0.032	0.422±0.037	0.404±0.028	0.402±0.026	0.005	[-0.004, 0.015]	0.808
OR_right	0.399±0.033	0.426±0.037	0.407±0.031	0.407±0.030	0.006	[-0.004, 0.016]	0.829
POPT_left	0.454±0.031	0.469±0.030	0.460±0.028	0.453±0.023	0.009	[-0.001, 0.018]	0.920
POPT_right	0.452±0.032	0.465±0.032	0.457±0.030	0.451±0.025	0.009	[-0.001, 0.019]	0.931
SCP_left	0.451±0.032	0.473±0.028	0.458±0.029	0.455±0.024	0.008	[-0.002, 0.018]	0.899
SCP_right	0.427±0.030	0.445±0.027	0.433±0.027	0.429±0.023	0.009	[-0.001, 0.018]	0.919
SLF_III_left	0.375±0.031	0.398±0.043	0.382±0.026	0.378±0.033	0.006	[-0.003, 0.016]	0.853
SLF_III_right	0.384±0.034	0.403±0.040	0.390±0.029	0.386±0.035	0.007	[-0.002, 0.017]	0.887
SLF_II_left	0.348±0.032	0.360±0.031	0.354±0.027	0.344±0.026	0.009	[-0.001, 0.019]	0.927
SLF_II_right	0.340±0.031	0.359±0.034	0.346±0.027	0.342±0.029	0.007	[-0.003, 0.016]	0.860
SLF_I_left	0.317±0.025	0.331±0.029	0.322±0.023	0.319±0.024	0.008	[-0.002, 0.018]	0.910
SLF_I_right	0.334±0.028	0.352±0.028	0.340±0.025	0.337±0.022	0.008	[-0.002, 0.018]	0.894
STR_left	0.483±0.043	0.503±0.040	0.489±0.041	0.485±0.034	0.007	[-0.003, 0.017]	0.876
STR_right	0.463±0.040	0.472±0.042	0.468±0.038	0.459±0.037	0.010	[0.000, 0.020]	0.941
UF_left	0.422±0.032	0.447±0.034	0.429±0.027	0.428±0.024	0.005	[-0.005, 0.015]	0.782
UF_right	0.417±0.031	0.439±0.035	0.423±0.028	0.422±0.026	0.005	[-0.004, 0.015]	0.815

[1] Units for raw and adjusted means, difference, and 95% HDI are arbitrary.

Data are presented as mean ± SD. Raw values are unadjusted sex-wise means. Adjusted values are sex-wise mean residual values after adjusting for age, education, intracranial volume, APOE e4 carriership, and family history of Alzheimer's disease. Bayesian model outputs include the mean difference and 95% highest density interval estimates as well as P+. P+ values approaching 1 indicate stronger evidence of female > male while P+ values approaching 0 indicate stronger evidence of male > female. The hierarchical model includes all tracts in a singular model and leverages partial pooling of the data, therefore the mean difference will not always be exactly equal to the difference in covariate adjusted means.

Supplemental table 4: Fractional anisotropy differences between women and men

	Raw means[1]		Covariate adjusted means[1]		Hierarchical Bayesian model[1]		
Tract	Females	Males	Females	Males	Mean Difference	95% HDI	P+
AF_left	0.387±0.015	0.385±0.017	0.387±0.015	0.386±0.016	0.001	[-0.004, 0.005]	0.608
AF_right	0.387±0.014	0.385±0.015	0.387±0.014	0.385±0.015	0.001	[-0.004, 0.005]	0.581
ATR_left	0.362±0.012	0.367±0.013	0.362±0.012	0.367±0.014	-0.003	[-0.007, 0.002]	0.197
ATR_right	0.344±0.011	0.345±0.014	0.343±0.011	0.345±0.014	-0.001	[-0.006, 0.004]	0.375
CA	0.393±0.046	0.396±0.052	0.393±0.046	0.396±0.052	-0.002	[-0.006, 0.003]	0.297
CC_1	0.435±0.018	0.435±0.023	0.435±0.017	0.436±0.022	0.001	[-0.004, 0.006]	0.652
CC_2	0.411±0.017	0.407±0.016	0.410±0.016	0.408±0.015	0.002	[-0.003, 0.007]	0.744
CC_3	0.403±0.016	0.400±0.016	0.403±0.015	0.400±0.016	0.002	[-0.003, 0.006]	0.721
CC_4	0.450±0.017	0.442±0.016	0.450±0.016	0.443±0.015	0.004	[-0.001, 0.009]	0.914
CC_5	0.451±0.018	0.442±0.023	0.451±0.018	0.443±0.021	0.005	[0.000, 0.010]	0.951
CC_6	0.479±0.014	0.477±0.017	0.479±0.014	0.477±0.016	0.001	[-0.004, 0.005]	0.620
CC_7	0.494±0.024	0.485±0.025	0.493±0.023	0.487±0.022	0.005	[0.000, 0.010]	0.947
CG_left	0.411±0.018	0.418±0.021	0.411±0.018	0.418±0.020	-0.004	[-0.008, 0.001]	0.116
CG_right	0.401±0.016	0.407±0.022	0.401±0.016	0.407±0.022	-0.002	[-0.007, 0.002]	0.207
CST_left	0.493±0.016	0.495±0.014	0.493±0.016	0.495±0.014	-0.002	[-0.007, 0.003]	0.231
CST_right	0.486±0.015	0.491±0.013	0.487±0.014	0.491±0.013	-0.003	[-0.008, 0.001]	0.131
FPT_left	0.458±0.014	0.465±0.014	0.458±0.014	0.464±0.014	-0.004	[-0.009, 0.001]	0.079
FPT_right	0.449±0.014	0.455±0.016	0.449±0.014	0.454±0.016	-0.004	[-0.009, 0.001]	0.086
FX_left	0.389±0.045	0.373±0.052	0.388±0.045	0.378±0.044	0.012	[0.007, 0.017]	1.000
FX_right	0.385±0.047	0.360±0.051	0.383±0.045	0.366±0.042	0.017	[0.012, 0.022]	1.000
ICP_left	0.414±0.014	0.421±0.019	0.414±0.014	0.421±0.019	-0.004	[-0.009, 0.001]	0.107
ICP_right	0.421±0.017	0.423±0.019	0.421±0.017	0.424±0.018	-0.002	[-0.007, 0.003]	0.257
IFO_left	0.420±0.014	0.417±0.016	0.419±0.014	0.417±0.014	0.001	[-0.003, 0.006]	0.669
IFO_right	0.426±0.015	0.424±0.016	0.426±0.015	0.425±0.015	0.001	[-0.004, 0.006]	0.636
ILF_left	0.412±0.017	0.408±0.019	0.412±0.017	0.409±0.017	0.002	[-0.003, 0.007]	0.745
ILF_right	0.411±0.016	0.409±0.014	0.411±0.016	0.410±0.013	0.000	[-0.004, 0.005]	0.520
MCP	0.480±0.013	0.483±0.016	0.480±0.012	0.483±0.016	-0.003	[-0.008, 0.002]	0.163
MLF_left	0.385±0.014	0.380±0.015	0.385±0.014	0.381±0.014	0.002	[-0.003, 0.007]	0.732
MLF_right	0.376±0.014	0.374±0.015	0.376±0.014	0.374±0.015	0.000	[-0.005, 0.005]	0.521
OR_left	0.421±0.017	0.418±0.016	0.421±0.016	0.418±0.015	0.001	[-0.004, 0.006]	0.633
OR_right	0.430±0.016	0.428±0.017	0.430±0.016	0.428±0.016	0.001	[-0.004, 0.005]	0.601
POPT_left	0.453±0.013	0.455±0.012	0.453±0.013	0.454±0.012	-0.002	[-0.007, 0.002]	0.220
POPT_right	0.459±0.013	0.463±0.013	0.459±0.013	0.463±0.013	-0.003	[-0.008, 0.002]	0.133
SCP_left	0.430±0.014	0.439±0.012	0.430±0.013	0.438±0.012	-0.007	[-0.012, -0.002]	0.010
SCP_right	0.409±0.014	0.420±0.017	0.410±0.013	0.419±0.015	-0.008	[-0.013, -0.003]	0.004
SLF_III_left	0.395±0.015	0.394±0.016	0.394±0.015	0.395±0.014	0.001	[-0.004, 0.005]	0.605
SLF_III_right	0.385±0.014	0.386±0.016	0.385±0.013	0.386±0.015	0.000	[-0.005, 0.005]	0.507
SLF_II_left	0.353±0.017	0.345±0.018	0.353±0.017	0.345±0.017	0.003	[-0.002, 0.008]	0.836
SLF_II_right	0.359±0.017	0.354±0.014	0.359±0.016	0.354±0.014	0.001	[-0.003, 0.006]	0.679
SLF_I_left	0.377±0.016	0.376±0.016	0.377±0.015	0.376±0.015	0.000	[-0.005, 0.005]	0.522
SLF_I_right	0.376±0.016	0.373±0.014	0.376±0.015	0.373±0.014	0.000	[-0.004, 0.005]	0.551
STR_left	0.396±0.018	0.398±0.016	0.396±0.018	0.397±0.016	-0.002	[-0.007, 0.003]	0.259
STR_right	0.398±0.017	0.402±0.018	0.398±0.017	0.402±0.018	-0.003	[-0.008, 0.002]	0.167
UF_left	0.363±0.014	0.367±0.020	0.362±0.014	0.368±0.020	-0.002	[-0.007, 0.003]	0.277
UF_right	0.353±0.013	0.354±0.017	0.353±0.013	0.355±0.017	0.000	[-0.005, 0.005]	0.462

[1] Units for raw and adjusted means, difference, and 95% HDI are arbitrary.

Data are presented as mean ± SD. Raw values are unadjusted sex-wise means. Adjusted values are sex-wise mean residual values after adjusting for age, education, APOE e4 carriership, and family history of Alzheimer's disease. Bayesian model outputs include the mean difference and 95% highest density interval estimates as well as P+. P+ indicates the proportion of the Bayesian samples (N = 80000) where the difference between women and men was > 0. P+ values approaching 1 indicate stronger evidence of female > male while P+ values approaching 0 indicate stronger evidence of male > female. The hierarchical model includes all tracts in a singular model and leverages partial pooling of the data, therefore the mean difference will not always be exactly equal to the difference in covariate adjusted means.

Supplemental table 5: Mean diffusivity differences between women and men

	Raw means[1]		Covariate adjusted means[1]		Hierarchical Bayesian model[1]		
Tract	Females	Males	Females	Males	Mean Difference	95% HDI	P+
AF_left	7.128±0.205	7.137±0.206	7.130±0.204	7.133±0.195	-0.004	[-0.095, 0.088]	0.476
AF_right	7.192±0.187	7.154±0.231	7.191±0.184	7.157±0.220	0.010	[-0.079, 0.104]	0.568
ATR_left	7.549±0.217	7.597±0.226	7.553±0.215	7.586±0.211	-0.031	[-0.121, 0.062]	0.294
ATR_right	7.821±0.205	7.890±0.245	7.826±0.203	7.877±0.228	-0.042	[-0.133, 0.049]	0.226
CA	8.130±0.359	8.088±0.375	8.136±0.359	8.071±0.355	-0.013	[-0.107, 0.081]	0.402
CC_1	8.136±0.326	8.070±0.326	8.136±0.321	8.070±0.328	-0.009	[-0.103, 0.085]	0.431
CC_2	8.451±0.362	8.512±0.263	8.452±0.360	8.508±0.259	-0.054	[-0.147, 0.039]	0.172
CC_3	8.723±0.409	8.867±0.349	8.727±0.405	8.856±0.339	-0.091	[-0.184, 0.003]	0.059
CC_4	8.374±0.366	8.526±0.340	8.382±0.366	8.503±0.306	-0.087	[-0.178, 0.005]	0.063
CC_5	8.786±0.421	9.014±0.562	8.804±0.419	8.967±0.496	-0.128	[-0.221, -0.036]	0.013
CC_6	7.888±0.263	7.957±0.311	7.895±0.261	7.937±0.283	-0.048	[-0.140, 0.042]	0.197
CC_7	9.113±0.658	9.498±0.796	9.134±0.644	9.442±0.765	-0.189	[-0.286, -0.090]	0.001
CG_left	7.273±0.194	7.232±0.243	7.271±0.191	7.239±0.237	0.011	[-0.080, 0.103]	0.577
CG_right	7.245±0.201	7.209±0.244	7.244±0.197	7.213±0.235	0.010	[-0.083, 0.101]	0.570
CST_left	6.954±0.160	6.958±0.127	6.953±0.159	6.960±0.121	0.007	[-0.085, 0.098]	0.552
CST_right	7.103±0.138	7.046±0.156	7.102±0.137	7.051±0.152	0.021	[-0.070, 0.112]	0.644
FPT_left	7.138±0.156	7.119±0.152	7.136±0.155	7.125±0.147	0.010	[-0.080, 0.102]	0.570
FPT_right	7.340±0.163	7.284±0.171	7.337±0.162	7.293±0.166	0.016	[-0.076, 0.107]	0.606
FX_left	15.978±1.810	16.751±2.067	16.062±1.739	16.525±1.816	-0.551	[-0.673, -0.433]	0.000
FX_right	15.773±1.790	16.777±1.990	15.871±1.680	16.510±1.653	-0.641	[-0.764, -0.521]	0.000
ICP_left	7.138±0.177	7.063±0.184	7.136±0.174	7.066±0.190	0.029	[-0.064, 0.120]	0.692
ICP_right	6.890±0.205	6.820±0.202	6.888±0.198	6.824±0.201	0.039	[-0.054, 0.131]	0.749
IFO_left	7.829±0.264	7.896±0.273	7.832±0.264	7.888±0.258	-0.040	[-0.130, 0.052]	0.240
IFO_right	7.863±0.266	7.954±0.321	7.870±0.263	7.936±0.305	-0.050	[-0.140, 0.040]	0.189
ILF_left	7.811±0.307	7.917±0.300	7.815±0.306	7.905±0.271	-0.052	[-0.144, 0.039]	0.179
ILF_right	7.706±0.284	7.828±0.299	7.713±0.277	7.810±0.285	-0.055	[-0.145, 0.038]	0.166
MCP	6.892±0.145	6.826±0.174	6.888±0.142	6.836±0.164	0.038	[-0.054, 0.130]	0.747
MLF_left	7.579±0.218	7.620±0.245	7.581±0.213	7.617±0.234	-0.024	[-0.115, 0.067]	0.337
MLF_right	7.403±0.210	7.373±0.274	7.403±0.209	7.374±0.257	0.000	[-0.091, 0.091]	0.499
OR_left	8.113±0.354	8.376±0.503	8.124±0.356	8.345±0.471	-0.109	[-0.204, -0.013]	0.029
OR_right	7.921±0.330	8.082±0.441	7.931±0.324	8.057±0.414	-0.081	[-0.173, 0.012]	0.078
POPT_left	7.635±0.174	7.692±0.183	7.638±0.172	7.684±0.171	-0.029	[-0.120, 0.062]	0.303
POPT_right	7.618±0.156	7.618±0.220	7.620±0.155	7.612±0.211	-0.012	[-0.104, 0.078]	0.416
SCP_left	8.028±0.220	8.081±0.229	8.026±0.216	8.087±0.228	-0.026	[-0.120, 0.068]	0.322
SCP_right	8.303±0.243	8.286±0.238	8.301±0.239	8.294±0.242	-0.016	[-0.112, 0.081]	0.386
SLF_III_left	7.091±0.225	7.104±0.216	7.093±0.224	7.098±0.200	-0.006	[-0.098, 0.087]	0.464
SLF_III_right	7.336±0.212	7.317±0.246	7.336±0.211	7.317±0.232	0.001	[-0.090, 0.091]	0.503
SLF_II_left	7.084±0.191	7.115±0.227	7.085±0.187	7.113±0.219	-0.008	[-0.101, 0.083]	0.447
SLF_II_right	7.208±0.188	7.201±0.239	7.207±0.183	7.205±0.232	0.000	[-0.091, 0.093]	0.504
SLF_I_left	7.282±0.216	7.289±0.255	7.284±0.209	7.284±0.249	-0.011	[-0.103, 0.081]	0.423
SLF_I_right	7.384±0.216	7.380±0.269	7.384±0.208	7.380±0.266	-0.008	[-0.099, 0.084]	0.446
STR_left	7.020±0.192	7.050±0.183	7.021±0.189	7.048±0.169	-0.007	[-0.100, 0.084]	0.454
STR_right	7.027±0.162	6.977±0.196	7.026±0.159	6.979±0.182	0.016	[-0.076, 0.107]	0.607
UF_left	7.675±0.236	7.562±0.227	7.673±0.234	7.567±0.227	0.021	[-0.073, 0.115]	0.632
UF_right	7.895±0.211	7.854±0.191	7.895±0.209	7.854±0.186	-0.005	[-0.098, 0.088]	0.460

[1] Units for raw and adjusted means, difference, and 95% HDI are 10^{-4} mm²/s.

Data are presented as mean ± SD. Raw values are unadjusted sex-wise means. Adjusted values are sex-wise mean residual values after adjusting for age, education, APOE e4 carriership, and family history of Alzheimer's disease. Bayesian model outputs include the mean difference and 95% highest density interval estimates as well as P+. P+ indicates the proportion of the Bayesian samples (N = 80000) where the difference between women and men was > 0. P+ values approaching 1 indicate stronger evidence of female > male while P+ values approaching 0 indicate stronger evidence of male > female. The hierarchical model includes all tracts in a singular model and leverages partial pooling of the data, therefore the mean difference will not always be exactly equal to the difference in covariate adjusted means.

Supplemental table 6: Fiber density differences between pre-menopausal women and age-matched men

	Raw means[1]		Covariate adjusted means[1]		Hierarchical Bayesian model[1]		
Tract	Females	Males	Females	Males	Mean Difference	95% HDI	P+
AF_left	0.368±0.014	0.360±0.014	0.368±0.013	0.361±0.012	0.009	[0.001, 0.017]	0.960
AF_right	0.392±0.016	0.381±0.016	0.391±0.016	0.382±0.012	0.011	[0.003, 0.019]	0.985
ATR_left	0.368±0.017	0.361±0.015	0.368±0.016	0.362±0.012	0.008	[0.000, 0.016]	0.948
ATR_right	0.366±0.016	0.358±0.013	0.366±0.016	0.358±0.012	0.008	[0.000, 0.016]	0.942
CA	0.447±0.025	0.440±0.022	0.447±0.025	0.439±0.021	0.007	[-0.001, 0.015]	0.909
CC_1	0.458±0.021	0.436±0.018	0.457±0.020	0.437±0.018	0.016	[0.008, 0.025]	0.999
CC_2	0.386±0.016	0.371±0.012	0.385±0.016	0.373±0.011	0.012	[0.004, 0.020]	0.989
CC_3	0.403±0.022	0.384±0.018	0.402±0.022	0.386±0.015	0.015	[0.007, 0.023]	0.998
CC_4	0.455±0.025	0.436±0.017	0.455±0.025	0.437±0.013	0.015	[0.007, 0.024]	0.999
CC_5	0.434±0.023	0.412±0.019	0.434±0.023	0.413±0.015	0.016	[0.008, 0.024]	0.999
CC_6	0.410±0.018	0.392±0.013	0.409±0.018	0.394±0.012	0.014	[0.006, 0.022]	0.997
CC_7	0.389±0.019	0.376±0.011	0.389±0.019	0.377±0.010	0.011	[0.002, 0.019]	0.982
CG_left	0.353±0.015	0.348±0.014	0.352±0.014	0.349±0.014	0.006	[-0.002, 0.014]	0.876
CG_right	0.365±0.017	0.358±0.013	0.364±0.017	0.360±0.012	0.008	[-0.001, 0.016]	0.931
CST_left	0.550±0.018	0.544±0.016	0.550±0.018	0.545±0.015	0.009	[0.001, 0.018]	0.963
CST_right	0.518±0.017	0.508±0.015	0.517±0.017	0.508±0.014	0.010	[0.002, 0.019]	0.977
FPT_left	0.476±0.016	0.470±0.016	0.475±0.016	0.471±0.015	0.008	[0.000, 0.016]	0.943
FPT_right	0.466±0.018	0.457±0.017	0.466±0.017	0.458±0.016	0.010	[0.002, 0.018]	0.972
FX_left	0.535±0.050	0.505±0.059	0.532±0.049	0.510±0.052	0.026	[0.018, 0.035]	1.000
FX_right	0.552±0.053	0.507±0.068	0.549±0.053	0.512±0.063	0.033	[0.023, 0.041]	1.000
ICP_left	0.415±0.016	0.411±0.017	0.415±0.015	0.411±0.015	0.006	[-0.003, 0.014]	0.866
ICP_right	0.380±0.017	0.371±0.016	0.379±0.016	0.371±0.014	0.009	[0.000, 0.017]	0.954
IFO_left	0.391±0.015	0.378±0.014	0.391±0.016	0.379±0.011	0.012	[0.003, 0.020]	0.988
IFO_right	0.388±0.016	0.373±0.011	0.387±0.016	0.374±0.009	0.012	[0.003, 0.020]	0.988
ILF_left	0.390±0.016	0.382±0.014	0.390±0.016	0.382±0.013	0.008	[0.000, 0.016]	0.949
ILF_right	0.396±0.016	0.385±0.016	0.395±0.016	0.386±0.013	0.010	[0.002, 0.018]	0.975
MCP	0.456±0.019	0.447±0.015	0.455±0.018	0.447±0.012	0.010	[0.002, 0.018]	0.973
MLF_left	0.379±0.015	0.363±0.012	0.378±0.014	0.365±0.011	0.012	[0.004, 0.020]	0.989
MLF_right	0.382±0.017	0.364±0.014	0.381±0.017	0.366±0.011	0.014	[0.005, 0.022]	0.996
OR_left	0.395±0.017	0.384±0.014	0.395±0.018	0.385±0.011	0.010	[0.002, 0.018]	0.977
OR_right	0.404±0.018	0.389±0.014	0.403±0.018	0.390±0.011	0.012	[0.004, 0.020]	0.991
POPT_left	0.465±0.015	0.450±0.015	0.464±0.014	0.451±0.015	0.013	[0.004, 0.021]	0.993
POPT_right	0.462±0.016	0.445±0.012	0.461±0.016	0.446±0.011	0.013	[0.005, 0.022]	0.995
SCP_left	0.462±0.016	0.463±0.015	0.462±0.016	0.463±0.014	0.005	[-0.004, 0.013]	0.815
SCP_right	0.438±0.015	0.435±0.014	0.437±0.015	0.435±0.013	0.006	[-0.002, 0.014]	0.877
SLF_III_left	0.368±0.016	0.363±0.015	0.368±0.016	0.364±0.012	0.008	[0.000, 0.016]	0.932
SLF_III_right	0.379±0.019	0.368±0.016	0.378±0.018	0.369±0.015	0.010	[0.002, 0.018]	0.975
SLF_II_left	0.339±0.017	0.325±0.013	0.339±0.017	0.325±0.013	0.011	[0.003, 0.019]	0.983
SLF_II_right	0.331±0.016	0.323±0.014	0.330±0.016	0.324±0.011	0.009	[0.001, 0.017]	0.955
SLF_I_left	0.311±0.014	0.306±0.015	0.311±0.013	0.306±0.014	0.005	[-0.003, 0.014]	0.850
SLF_I_right	0.326±0.015	0.323±0.011	0.326±0.015	0.324±0.010	0.005	[-0.003, 0.014]	0.849
STR_left	0.495±0.020	0.485±0.017	0.494±0.020	0.486±0.016	0.010	[0.002, 0.018]	0.976
STR_right	0.473±0.019	0.451±0.016	0.472±0.018	0.452±0.016	0.017	[0.009, 0.025]	0.999
UF_left	0.430±0.019	0.420±0.015	0.429±0.019	0.420±0.015	0.010	[0.002, 0.018]	0.970
UF_right	0.418±0.019	0.408±0.014	0.418±0.019	0.409±0.014	0.009	[0.001, 0.018]	0.969

[1] Units for raw and adjusted means, difference, and 95% HDI are arbitrary.

Data are presented as mean ± SD. Raw values are unadjusted sex-wise means. Adjusted values are sex-wise mean residual values after adjusting for age, education, APOE e4 carriership, and family history of Alzheimer's disease. Bayesian model outputs include the mean difference and 95% highest density interval estimates as well as P+. P+ indicates the proportion of the Bayesian samples (N = 80000) where the difference between women and men was > 0. P+ values approaching 1 indicate stronger evidence of female > male while P+ values approaching 0 indicate stronger evidence of male > female. The hierarchical model includes all tracts in a singular model and leverages partial pooling of the data, therefore the mean difference will not always be exactly equal to the difference in covariate adjusted means.

Supplemental table 7: Fiber cross-section differences between pre-menopausal women and age-matched men

	Raw means[1]		Covariate adjusted means[1]		Hierarchical Bayesian model[1]		
Tract	Females	Males	Females	Males	Mean Difference	95% HDI	P+
AF_left	0.017±0.058	0.111±0.067	0.051±0.029	0.047±0.034	0.002	[-0.023, 0.026]	0.549
AF_right	0.012±0.059	0.109±0.068	0.046±0.033	0.044±0.039	0.000	[-0.025, 0.024]	0.490
ATR_left	-0.009±0.077	0.084±0.077	0.029±0.043	0.012±0.044	0.011	[-0.014, 0.037]	0.761
ATR_right	-0.003±0.079	0.089±0.089	0.036±0.049	0.016±0.054	0.012	[-0.013, 0.038]	0.779
CA	-0.001±0.066	0.094±0.082	0.030±0.057	0.037±0.062	-0.008	[-0.033, 0.017]	0.301
CC_1	-0.012±0.074	0.058±0.093	0.021±0.048	-0.004±0.064	0.023	[-0.003, 0.049]	0.924
CC_2	0.004±0.073	0.086±0.078	0.039±0.047	0.020±0.044	0.014	[-0.011, 0.040]	0.814
CC_3	0.019±0.094	0.110±0.096	0.056±0.073	0.040±0.061	0.009	[-0.016, 0.035]	0.718
CC_4	-0.014±0.069	0.069±0.065	0.013±0.057	0.018±0.050	0.000	[-0.025, 0.025]	0.497
CC_5	-0.019±0.056	0.062±0.061	0.009±0.040	0.010±0.046	0.003	[-0.021, 0.028]	0.581
CC_6	-0.026±0.050	0.078±0.072	0.007±0.033	0.016±0.046	-0.007	[-0.032, 0.018]	0.318
CC_7	0.008±0.068	0.160±0.082	0.049±0.063	0.083±0.054	-0.036	[-0.064, -0.008]	0.017
CG_left	-0.030±0.060	0.064±0.076	0.004±0.032	0.000±0.044	0.006	[-0.019, 0.031]	0.643
CG_right	-0.020±0.060	0.059±0.075	0.011±0.032	0.001±0.041	0.011	[-0.014, 0.037]	0.763
CST_left	-0.010±0.067	0.043±0.061	0.011±0.055	0.003±0.054	0.013	[-0.012, 0.038]	0.799
CST_right	-0.022±0.068	0.045±0.068	0.002±0.056	-0.001±0.051	0.007	[-0.017, 0.032]	0.683
FPT_left	-0.013±0.057	0.059±0.059	0.014±0.041	0.008±0.035	0.009	[-0.016, 0.033]	0.717
FPT_right	-0.006±0.064	0.055±0.059	0.021±0.046	0.005±0.028	0.016	[-0.009, 0.041]	0.851
FX_left	-0.095±0.105	0.051±0.102	-0.046±0.077	-0.040±0.076	-0.012	[-0.039, 0.015]	0.247
FX_right	-0.095±0.098	0.058±0.112	-0.046±0.077	-0.035±0.082	-0.019	[-0.046, 0.008]	0.135
ICP_left	-0.038±0.064	0.021±0.050	-0.018±0.050	-0.016±0.054	0.004	[-0.022, 0.029]	0.589
ICP_right	-0.031±0.060	0.012±0.058	-0.012±0.044	-0.023±0.054	0.014	[-0.012, 0.040]	0.811
IFO_left	0.011±0.056	0.118±0.073	0.048±0.033	0.049±0.027	-0.005	[-0.030, 0.020]	0.384
IFO_right	0.000±0.054	0.132±0.071	0.038±0.040	0.059±0.041	-0.022	[-0.047, 0.004]	0.087
ILF_left	0.033±0.055	0.144±0.077	0.068±0.044	0.077±0.029	-0.013	[-0.038, 0.013]	0.206
ILF_right	0.015±0.063	0.143±0.079	0.054±0.048	0.070±0.042	-0.018	[-0.043, 0.008]	0.126
MCP	-0.015±0.060	0.031±0.063	0.004±0.047	-0.004±0.056	0.011	[-0.015, 0.036]	0.752
MLF_left	0.001±0.054	0.098±0.066	0.033±0.032	0.037±0.040	-0.004	[-0.029, 0.021]	0.403
MLF_right	-0.011±0.050	0.092±0.074	0.021±0.034	0.031±0.045	-0.008	[-0.033, 0.017]	0.309
OR_left	0.019±0.070	0.133±0.091	0.057±0.057	0.063±0.047	-0.011	[-0.036, 0.014]	0.242
OR_right	-0.016±0.068	0.140±0.074	0.024±0.062	0.064±0.056	-0.039	[-0.066, -0.010]	0.012
POPT_left	-0.024±0.053	0.053±0.059	0.004±0.030	0.000±0.039	0.005	[-0.019, 0.030]	0.629
POPT_right	-0.029±0.050	0.051±0.063	-0.002±0.032	0.000±0.046	0.002	[-0.023, 0.026]	0.546
SCP_left	-0.033±0.056	0.021±0.053	-0.011±0.042	-0.019±0.044	0.013	[-0.013, 0.037]	0.790
SCP_right	-0.033±0.055	0.021±0.053	-0.012±0.041	-0.018±0.044	0.011	[-0.014, 0.036]	0.760
SLF_III_left	0.008±0.065	0.112±0.096	0.045±0.042	0.043±0.064	0.001	[-0.024, 0.025]	0.518
SLF_III_right	0.017±0.063	0.098±0.078	0.048±0.039	0.039±0.058	0.008	[-0.017, 0.033]	0.702
SLF_II_left	0.030±0.065	0.105±0.069	0.060±0.039	0.049±0.044	0.011	[-0.015, 0.035]	0.762
SLF_II_right	0.021±0.075	0.118±0.075	0.055±0.051	0.053±0.044	0.003	[-0.022, 0.028]	0.567
SLF_I_left	0.010±0.054	0.081±0.062	0.035±0.041	0.034±0.041	0.006	[-0.019, 0.030]	0.644
SLF_I_right	0.001±0.063	0.092±0.063	0.031±0.046	0.035±0.040	0.000	[-0.025, 0.024]	0.493
STR_left	-0.028±0.059	0.044±0.068	-0.005±0.049	0.001±0.058	0.004	[-0.021, 0.029]	0.601
STR_right	-0.013±0.057	0.046±0.072	0.009±0.046	0.004±0.055	0.012	[-0.013, 0.037]	0.775
UF_left	-0.003±0.055	0.095±0.061	0.029±0.039	0.035±0.026	-0.004	[-0.028, 0.021]	0.407
UF_right	0.005±0.058	0.096±0.072	0.038±0.037	0.034±0.040	0.002	[-0.023, 0.026]	0.544

[1] Units for raw and adjusted means, difference, and 95% HDI are arbitrary.

Data are presented as mean ± SD. Raw values are unadjusted sex-wise means. Adjusted values are sex-wise mean residual values after adjusting for age, education, intracranial volume, APOE e4 carriership, and family history of Alzheimer's disease. Bayesian model outputs include the mean difference and 95% highest density interval estimates as well as P+. P+ values approaching 1 indicate stronger evidence of female > male while P+ values approaching 0 indicate stronger evidence of male > female. The hierarchical model includes all tracts in a singular model and leverages partial pooling of the data, therefore the mean difference will not always be exactly equal to the difference in covariate adjusted means.

Supplemental table 8: FDC differences between pre-menopausal women and age-matched men

	Raw means[1]		Covariate adjusted means[1]		Hierarchical Bayesian model[1]		
Tract	Females	Males	Females	Males	Mean Difference	95% HDI	P+
AF_left	0.378±0.026	0.406±0.035	0.389±0.020	0.385±0.023	0.004	[-0.013, 0.021]	0.658
AF_right	0.399±0.026	0.427±0.038	0.409±0.022	0.407±0.026	0.003	[-0.014, 0.020]	0.620
ATR_left	0.366±0.028	0.393±0.033	0.378±0.019	0.370±0.020	0.006	[-0.011, 0.023]	0.711
ATR_right	0.365±0.028	0.392±0.040	0.377±0.022	0.369±0.029	0.006	[-0.011, 0.023]	0.718
CA	0.447±0.041	0.484±0.048	0.457±0.041	0.465±0.044	-0.003	[-0.021, 0.015]	0.399
CC_1	0.455±0.039	0.461±0.052	0.463±0.032	0.445±0.047	0.018	[0.000, 0.036]	0.948
CC_2	0.389±0.031	0.405±0.037	0.398±0.027	0.388±0.027	0.010	[-0.007, 0.027]	0.834
CC_3	0.412±0.047	0.430±0.045	0.422±0.042	0.411±0.037	0.012	[-0.005, 0.030]	0.871
CC_4	0.453±0.046	0.469±0.035	0.458±0.045	0.459±0.032	0.008	[-0.010, 0.025]	0.757
CC_5	0.428±0.036	0.440±0.037	0.433±0.035	0.431±0.034	0.008	[-0.009, 0.025]	0.781
CC_6	0.402±0.027	0.428±0.036	0.410±0.027	0.414±0.031	0.001	[-0.016, 0.019]	0.548
CC_7	0.392±0.034	0.440±0.038	0.404±0.036	0.417±0.028	-0.009	[-0.027, 0.009]	0.215
CG_left	0.344±0.024	0.375±0.036	0.354±0.020	0.356±0.029	0.001	[-0.016, 0.018]	0.544
CG_right	0.360±0.025	0.384±0.033	0.368±0.022	0.368±0.024	0.003	[-0.014, 0.020]	0.630
CST_left	0.548±0.044	0.567±0.039	0.557±0.040	0.550±0.034	0.010	[-0.007, 0.027]	0.824
CST_right	0.510±0.041	0.531±0.037	0.520±0.038	0.513±0.031	0.009	[-0.008, 0.026]	0.801
FPT_left	0.471±0.036	0.497±0.036	0.483±0.031	0.476±0.025	0.007	[-0.010, 0.024]	0.758
FPT_right	0.464±0.036	0.482±0.037	0.474±0.032	0.463±0.025	0.011	[-0.006, 0.029]	0.854
FX_left	0.484±0.037	0.524±0.051	0.495±0.035	0.504±0.046	-0.004	[-0.022, 0.014]	0.358
FX_right	0.500±0.041	0.526±0.057	0.509±0.038	0.509±0.052	0.003	[-0.015, 0.020]	0.595
ICP_left	0.402±0.029	0.422±0.023	0.408±0.025	0.411±0.024	0.004	[-0.014, 0.022]	0.637
ICP_right	0.370±0.027	0.376±0.025	0.375±0.024	0.366±0.024	0.012	[-0.006, 0.029]	0.858
IFO_left	0.395±0.025	0.423±0.034	0.406±0.022	0.402±0.018	0.004	[-0.013, 0.021]	0.640
IFO_right	0.387±0.024	0.421±0.033	0.398±0.024	0.401±0.022	-0.001	[-0.017, 0.017]	0.481
ILF_left	0.403±0.028	0.439±0.036	0.415±0.028	0.418±0.021	-0.002	[-0.019, 0.015]	0.424
ILF_right	0.402±0.034	0.441±0.040	0.415±0.032	0.417±0.026	-0.001	[-0.018, 0.016]	0.455
MCP	0.454±0.035	0.465±0.032	0.459±0.032	0.454±0.029	0.010	[-0.007, 0.027]	0.822
MLF_left	0.381±0.026	0.403±0.031	0.389±0.023	0.388±0.025	0.004	[-0.013, 0.021]	0.657
MLF_right	0.380±0.026	0.403±0.036	0.387±0.025	0.388±0.027	0.002	[-0.015, 0.019]	0.586
OR_left	0.400±0.032	0.434±0.042	0.412±0.031	0.412±0.024	0.000	[-0.017, 0.017]	0.506
OR_right	0.396±0.028	0.442±0.036	0.407±0.030	0.420±0.026	-0.008	[-0.026, 0.010]	0.233
POPT_left	0.456±0.028	0.474±0.033	0.464±0.023	0.458±0.027	0.008	[-0.009, 0.025]	0.778
POPT_right	0.451±0.030	0.470±0.037	0.460±0.027	0.453±0.030	0.008	[-0.010, 0.024]	0.763
SCP_left	0.448±0.033	0.473±0.028	0.458±0.028	0.455±0.025	0.006	[-0.012, 0.022]	0.708
SCP_right	0.425±0.030	0.445±0.029	0.433±0.026	0.428±0.024	0.007	[-0.010, 0.024]	0.746
SLF_III_left	0.375±0.027	0.410±0.049	0.388±0.023	0.387±0.035	0.001	[-0.016, 0.019]	0.550
SLF_III_right	0.387±0.032	0.410±0.046	0.398±0.026	0.390±0.038	0.008	[-0.009, 0.026]	0.775
SLF_II_left	0.354±0.031	0.366±0.032	0.362±0.026	0.352±0.026	0.011	[-0.007, 0.028]	0.842
SLF_II_right	0.342±0.032	0.369±0.039	0.352±0.026	0.350±0.028	0.004	[-0.014, 0.021]	0.632
SLF_I_left	0.318±0.025	0.336±0.029	0.325±0.021	0.323±0.025	0.006	[-0.011, 0.023]	0.721
SLF_I_right	0.332±0.029	0.360±0.028	0.341±0.027	0.343±0.022	0.001	[-0.016, 0.019]	0.557
STR_left	0.486±0.038	0.506±0.042	0.494±0.036	0.490±0.036	0.008	[-0.009, 0.025]	0.773
STR_right	0.467±0.036	0.471±0.042	0.473±0.033	0.461±0.037	0.015	[-0.002, 0.033]	0.918
UF_left	0.429±0.032	0.461±0.031	0.440±0.028	0.440±0.021	0.002	[-0.015, 0.019]	0.591
UF_right	0.420±0.031	0.449±0.035	0.430±0.030	0.430±0.026	0.002	[-0.015, 0.019]	0.588

[1] Units for raw and adjusted means, difference, and 95% HDI are arbitrary.

Data are presented as mean ± SD. Raw values are unadjusted sex-wise means. Adjusted values are sex-wise mean residual values after adjusting for age, education, intracranial volume, APOE e4 carriership, and family history of Alzheimer's disease. Bayesian model outputs include the mean difference and 95% highest density interval estimates as well as P+. P+ values approaching 1 indicate stronger evidence of female > male while P+ values approaching 0 indicate stronger evidence of male > female. The hierarchical model includes all tracts in a singular model and leverages partial pooling of the data, therefore the mean difference will not always be exactly equal to the difference in covariate adjusted means.

Supplemental table 9: Fractional anisotropy differences between pre-menopausal women and age-matched men

	Raw means[1]		Covariate adjusted means[1]		Hierarchical Bayesian model[1]		
Tract	Females	Males	Females	Males	Mean Difference	95% HDI	P+
AF_left	0.388±0.014	0.393±0.014	0.387±0.012	0.393±0.013	-0.003	[-0.010, 0.003]	0.205
AF_right	0.388±0.014	0.389±0.014	0.387±0.013	0.390±0.011	-0.002	[-0.008, 0.004]	0.326
ATR_left	0.363±0.010	0.369±0.013	0.363±0.010	0.370±0.013	-0.004	[-0.011, 0.002]	0.153
ATR_right	0.343±0.009	0.346±0.014	0.343±0.009	0.347±0.014	-0.003	[-0.010, 0.003]	0.224
CA	0.397±0.052	0.406±0.046	0.400±0.048	0.402±0.047	-0.008	[-0.015, -0.001]	0.035
CC_1	0.437±0.019	0.442±0.021	0.436±0.018	0.442±0.019	-0.003	[-0.009, 0.003]	0.228
CC_2	0.413±0.014	0.411±0.014	0.412±0.013	0.412±0.012	-0.001	[-0.007, 0.005]	0.393
CC_3	0.406±0.016	0.402±0.013	0.405±0.014	0.403±0.012	0.000	[-0.007, 0.006]	0.448
CC_4	0.451±0.017	0.446±0.015	0.450±0.017	0.447±0.014	0.000	[-0.007, 0.006]	0.461
CC_5	0.454±0.018	0.449±0.018	0.454±0.018	0.450±0.017	0.000	[-0.006, 0.007]	0.519
CC_6	0.480±0.016	0.480±0.018	0.480±0.015	0.480±0.016	-0.002	[-0.008, 0.005]	0.343
CC_7	0.496±0.026	0.496±0.024	0.496±0.026	0.496±0.024	-0.002	[-0.009, 0.004]	0.307
CG_left	0.414±0.018	0.423±0.017	0.413±0.016	0.424±0.015	-0.004	[-0.011, 0.002]	0.137
CG_right	0.404±0.014	0.414±0.018	0.403±0.013	0.415±0.017	-0.004	[-0.011, 0.002]	0.133
CST_left	0.491±0.014	0.496±0.013	0.492±0.014	0.496±0.011	-0.004	[-0.011, 0.002]	0.158
CST_right	0.485±0.012	0.490±0.012	0.485±0.011	0.489±0.011	-0.004	[-0.010, 0.003]	0.174
FPT_left	0.457±0.014	0.465±0.014	0.458±0.013	0.464±0.013	-0.005	[-0.011, 0.002]	0.114
FPT_right	0.448±0.012	0.454±0.017	0.448±0.011	0.454±0.016	-0.004	[-0.011, 0.002]	0.143
FX_left	0.397±0.037	0.386±0.043	0.394±0.035	0.390±0.036	0.005	[-0.002, 0.012]	0.865
FX_right	0.399±0.040	0.375±0.044	0.397±0.039	0.379±0.041	0.007	[0.000, 0.015]	0.944
ICP_left	0.415±0.013	0.426±0.021	0.415±0.012	0.425±0.020	-0.006	[-0.012, 0.001]	0.071
ICP_right	0.422±0.019	0.425±0.020	0.422±0.017	0.424±0.020	-0.004	[-0.010, 0.002]	0.158
IFO_left	0.420±0.016	0.424±0.013	0.420±0.015	0.423±0.013	-0.003	[-0.010, 0.003]	0.191
IFO_right	0.427±0.017	0.430±0.014	0.427±0.016	0.430±0.013	-0.003	[-0.009, 0.003]	0.215
ILF_left	0.412±0.019	0.415±0.015	0.412±0.018	0.415±0.015	-0.004	[-0.010, 0.002]	0.169
ILF_right	0.411±0.017	0.411±0.015	0.411±0.016	0.411±0.015	-0.002	[-0.009, 0.004]	0.269
MCP	0.479±0.011	0.485±0.016	0.479±0.010	0.485±0.015	-0.004	[-0.010, 0.003]	0.184
MLF_left	0.386±0.016	0.384±0.014	0.386±0.014	0.385±0.014	-0.001	[-0.007, 0.005]	0.394
MLF_right	0.376±0.014	0.378±0.017	0.376±0.012	0.378±0.015	-0.002	[-0.009, 0.004]	0.276
OR_left	0.421±0.016	0.422±0.017	0.421±0.016	0.422±0.016	-0.002	[-0.009, 0.004]	0.277
OR_right	0.431±0.015	0.431±0.016	0.431±0.015	0.432±0.015	-0.002	[-0.008, 0.005]	0.339
POPT_left	0.452±0.012	0.456±0.013	0.452±0.012	0.455±0.012	-0.003	[-0.010, 0.003]	0.202
POPT_right	0.458±0.013	0.463±0.014	0.458±0.012	0.463±0.012	-0.004	[-0.010, 0.003]	0.185
SCP_left	0.428±0.015	0.435±0.012	0.428±0.013	0.434±0.013	-0.005	[-0.012, 0.001]	0.088
SCP_right	0.406±0.015	0.415±0.015	0.407±0.012	0.414±0.013	-0.006	[-0.012, 0.001]	0.075
SLF_III_left	0.396±0.016	0.402±0.013	0.396±0.014	0.403±0.012	-0.003	[-0.010, 0.003]	0.199
SLF_III_right	0.387±0.013	0.391±0.014	0.386±0.012	0.391±0.013	-0.003	[-0.009, 0.003]	0.232
SLF_II_left	0.355±0.018	0.346±0.017	0.354±0.017	0.347±0.017	0.001	[-0.005, 0.008]	0.609
SLF_II_right	0.359±0.018	0.356±0.013	0.359±0.015	0.357±0.013	0.000	[-0.007, 0.006]	0.468
SLF_I_left	0.380±0.016	0.377±0.016	0.379±0.015	0.378±0.016	-0.001	[-0.007, 0.006]	0.409
SLF_I_right	0.376±0.017	0.373±0.013	0.376±0.016	0.374±0.012	-0.001	[-0.007, 0.006]	0.440
STR_left	0.397±0.017	0.400±0.014	0.397±0.017	0.400±0.013	-0.003	[-0.009, 0.003]	0.227
STR_right	0.400±0.015	0.401±0.015	0.400±0.015	0.401±0.015	-0.002	[-0.009, 0.004]	0.280
UF_left	0.363±0.015	0.377±0.014	0.363±0.014	0.376±0.014	-0.007	[-0.014, 0.000]	0.049
UF_right	0.354±0.014	0.360±0.014	0.354±0.013	0.360±0.013	-0.005	[-0.011, 0.002]	0.130

[1] Units for raw and adjusted means, difference, and 95% HDI are arbitrary.

Data are presented as mean ± SD. Raw values are unadjusted sex-wise means. Adjusted values are sex-wise mean residual values after adjusting for age, education, APOE e4 carriership, and family history of Alzheimer's disease. Bayesian model outputs include the mean difference and 95% highest density interval estimates as well as P+. P+ indicates the proportion of the Bayesian samples (N = 80000) where the difference between women and men was > 0. P+ values approaching 1 indicate stronger evidence of female > male while P+ values approaching 0 indicate stronger evidence of male > female. The hierarchical model includes all tracts in a singular model and leverages partial pooling of the data, therefore the mean difference will not always be exactly equal to the difference in covariate adjusted means.

Supplemental table 10: Mean diffusivity differences between pre-menopausal women and age-matched men

	Raw means[1]		Covariate adjusted means[1]		Hierarchical Bayesian model[1]		
Tract	Females	Males	Females	Males	Mean Difference	95% HDI	P+
AF_left	7.113±0.219	7.055±0.182	7.112±0.215	7.058±0.172	0.062	[-0.075, 0.199]	0.768
AF_right	7.195±0.183	7.109±0.214	7.194±0.176	7.111±0.203	0.063	[-0.071, 0.202]	0.772
ATR_left	7.530±0.228	7.511±0.213	7.529±0.227	7.512±0.208	0.025	[-0.108, 0.167]	0.617
ATR_right	7.809±0.194	7.828±0.273	7.812±0.198	7.821±0.246	-0.002	[-0.137, 0.135]	0.487
CA	8.101±0.369	7.932±0.293	8.081±0.340	7.971±0.273	0.018	[-0.125, 0.159]	0.573
CC_1	8.154±0.371	8.046±0.325	8.153±0.361	8.047±0.331	0.002	[-0.136, 0.139]	0.503
CC_2	8.449±0.363	8.463±0.267	8.451±0.350	8.458±0.271	-0.046	[-0.180, 0.093]	0.289
CC_3	8.697±0.469	8.839±0.383	8.710±0.452	8.816±0.386	-0.090	[-0.225, 0.048]	0.146
CC_4	8.369±0.428	8.424±0.342	8.370±0.419	8.423±0.347	-0.051	[-0.187, 0.086]	0.275
CC_5	8.728±0.456	8.800±0.396	8.727±0.456	8.802±0.367	-0.079	[-0.217, 0.058]	0.177
CC_6	7.851±0.312	7.847±0.301	7.848±0.304	7.853±0.281	-0.002	[-0.141, 0.131]	0.491
CC_7	9.080±0.681	9.400±0.869	9.084±0.663	9.393±0.882	-0.160	[-0.307, -0.023]	0.034
CG_left	7.265±0.202	7.183±0.211	7.260±0.194	7.192±0.198	0.056	[-0.077, 0.195]	0.747
CG_right	7.250±0.193	7.159±0.263	7.249±0.184	7.161±0.244	0.060	[-0.076, 0.197]	0.763
CST_left	6.970±0.184	6.920±0.124	6.966±0.174	6.928±0.124	0.069	[-0.068, 0.206]	0.794
CST_right	7.112±0.129	7.022±0.172	7.108±0.121	7.030±0.161	0.069	[-0.068, 0.204]	0.793
FPT_left	7.159±0.156	7.096±0.133	7.155±0.150	7.103±0.129	0.060	[-0.078, 0.196]	0.760
FPT_right	7.362±0.150	7.275±0.168	7.358±0.143	7.281±0.166	0.051	[-0.084, 0.188]	0.727
FX_left	15.597±1.580	16.327±1.783	15.679±1.542	16.173±1.574	-0.676	[-0.855, -0.499]	0.000
FX_right	15.218±1.504	16.309±1.865	15.286±1.500	16.183±1.731	-0.738	[-0.914, -0.560]	0.000
ICP_left	7.167±0.177	7.011±0.193	7.156±0.163	7.031±0.197	0.079	[-0.059, 0.218]	0.821
ICP_right	6.932±0.235	6.802±0.231	6.919±0.216	6.828±0.217	0.088	[-0.050, 0.225]	0.850
IFO_left	7.826±0.328	7.794±0.258	7.818±0.318	7.808±0.241	0.005	[-0.135, 0.139]	0.524
IFO_right	7.872±0.273	7.897±0.236	7.873±0.255	7.896±0.218	-0.010	[-0.146, 0.124]	0.453
ILF_left	7.800±0.387	7.821±0.287	7.792±0.370	7.836±0.257	-0.006	[-0.143, 0.130]	0.473
ILF_right	7.705±0.285	7.795±0.202	7.706±0.255	7.793±0.197	-0.012	[-0.148, 0.125]	0.445
MCP	6.925±0.129	6.864±0.163	6.925±0.132	6.862±0.144	0.076	[-0.059, 0.215]	0.813
MLF_left	7.572±0.263	7.541±0.218	7.569±0.252	7.547±0.215	0.024	[-0.112, 0.159]	0.611
MLF_right	7.413±0.236	7.300±0.253	7.411±0.217	7.303±0.220	0.054	[-0.084, 0.190]	0.734
OR_left	8.135±0.448	8.237±0.465	8.133±0.444	8.239±0.441	-0.047	[-0.183, 0.091]	0.291
OR_right	7.912±0.293	8.009±0.347	7.915±0.273	8.004±0.346	-0.028	[-0.167, 0.106]	0.375
POPT_left	7.626±0.184	7.631±0.187	7.623±0.178	7.637±0.164	0.011	[-0.124, 0.149]	0.555
POPT_right	7.610±0.177	7.558±0.226	7.606±0.169	7.565±0.200	0.026	[-0.110, 0.162]	0.620
SCP_left	8.065±0.222	8.109±0.269	8.058±0.203	8.122±0.264	-0.029	[-0.164, 0.110]	0.367
SCP_right	8.337±0.229	8.303±0.284	8.325±0.196	8.326±0.279	-0.030	[-0.167, 0.112]	0.361
SLF_III_left	7.070±0.242	7.014±0.193	7.069±0.237	7.018±0.176	0.064	[-0.074, 0.201]	0.778
SLF_III_right	7.324±0.195	7.261±0.233	7.321±0.189	7.267±0.221	0.048	[-0.086, 0.186]	0.716
SLF_II_left	7.062±0.203	7.063±0.209	7.065±0.194	7.058±0.202	0.053	[-0.084, 0.191]	0.734
SLF_II_right	7.210±0.183	7.154±0.241	7.211±0.169	7.153±0.230	0.055	[-0.079, 0.193]	0.742
SLF_I_left	7.229±0.201	7.228±0.249	7.230±0.186	7.226±0.234	0.041	[-0.095, 0.179]	0.688
SLF_I_right	7.365±0.213	7.319±0.265	7.364±0.195	7.320±0.255	0.042	[-0.094, 0.180]	0.688
STR_left	6.994±0.178	6.997±0.178	6.989±0.173	7.006±0.145	0.055	[-0.084, 0.191]	0.743
STR_right	7.015±0.147	6.938±0.195	7.014±0.142	6.939±0.179	0.074	[-0.065, 0.210]	0.804
UF_left	7.678±0.276	7.474±0.210	7.666±0.272	7.497±0.176	0.054	[-0.087, 0.192]	0.732
UF_right	7.911±0.219	7.798±0.170	7.910±0.216	7.801±0.169	0.020	[-0.119, 0.156]	0.589

[1] Units for raw and adjusted means, difference, and 95% HDI are 10^{-4} mm²/s.

Data are presented as mean ± SD. Raw values are unadjusted sex-wise means. Adjusted values are sex-wise mean residual values after adjusting for age, education, APOE e4 carriership, and family history of Alzheimer's disease. Bayesian model outputs include the mean difference and 95% highest density interval estimates as well as P+. P+ indicates the proportion of the Bayesian samples (N = 80000) where the difference between women and men was > 0. P+ values approaching 1 indicate stronger evidence of female > male while P+ values approaching 0 indicate stronger evidence of male > female. The hierarchical model includes all tracts in a singular model and leverages partial pooling of the data, therefore the mean difference will not always be exactly equal to the difference in covariate adjusted means.

Supplemental table 11: Fiber density differences between peri-menopausal women and age-matched men

	Raw means[1]		Covariate adjusted means[1]		Hierarchical Bayesian model[1]		
Tract	Females	Males	Females	Males	Mean Difference	95% HDI	P+
AF_left	0.368±0.016	0.359±0.013	0.367±0.015	0.361±0.012	0.008	[0.001, 0.015]	0.967
AF_right	0.393±0.020	0.382±0.016	0.392±0.020	0.384±0.014	0.008	[0.001, 0.016]	0.970
ATR_left	0.366±0.017	0.361±0.016	0.365±0.015	0.362±0.012	0.008	[0.001, 0.015]	0.962
ATR_right	0.367±0.015	0.360±0.015	0.366±0.014	0.362±0.014	0.008	[0.001, 0.015]	0.958
CA	0.441±0.028	0.439±0.022	0.440±0.026	0.442±0.023	0.007	[0.000, 0.015]	0.942
CC_1	0.455±0.020	0.436±0.017	0.453±0.019	0.438±0.016	0.010	[0.002, 0.017]	0.982
CC_2	0.384±0.016	0.372±0.014	0.384±0.015	0.374±0.012	0.009	[0.002, 0.016]	0.974
CC_3	0.400±0.021	0.386±0.020	0.399±0.018	0.389±0.017	0.009	[0.002, 0.017]	0.980
CC_4	0.458±0.023	0.439±0.019	0.456±0.022	0.444±0.015	0.010	[0.002, 0.017]	0.984
CC_5	0.433±0.022	0.414±0.019	0.431±0.021	0.419±0.014	0.010	[0.003, 0.017]	0.986
CC_6	0.411±0.019	0.393±0.014	0.409±0.018	0.396±0.013	0.010	[0.002, 0.017]	0.984
CC_7	0.385±0.018	0.375±0.012	0.384±0.017	0.378±0.011	0.008	[0.001, 0.016]	0.970
CG_left	0.351±0.014	0.348±0.013	0.351±0.014	0.347±0.012	0.007	[0.000, 0.014]	0.936
CG_right	0.365±0.018	0.357±0.012	0.365±0.017	0.358±0.012	0.008	[0.001, 0.015]	0.961
CST_left	0.555±0.021	0.548±0.019	0.554±0.021	0.549±0.017	0.007	[-0.001, 0.014]	0.926
CST_right	0.520±0.021	0.513±0.019	0.519±0.021	0.515±0.018	0.007	[0.000, 0.015]	0.943
FPT_left	0.478±0.017	0.474±0.018	0.478±0.016	0.473±0.016	0.007	[-0.001, 0.014]	0.925
FPT_right	0.467±0.015	0.462±0.020	0.467±0.015	0.463±0.019	0.007	[0.000, 0.014]	0.935
FX_left	0.519±0.068	0.499±0.070	0.515±0.058	0.506±0.059	0.013	[0.004, 0.021]	0.993
FX_right	0.536±0.066	0.508±0.072	0.531±0.055	0.516±0.064	0.014	[0.005, 0.022]	0.996
ICP_left	0.418±0.019	0.412±0.016	0.417±0.019	0.413±0.016	0.007	[0.000, 0.014]	0.944
ICP_right	0.382±0.018	0.372±0.017	0.381±0.018	0.372±0.016	0.008	[0.001, 0.015]	0.963
IFO_left	0.388±0.016	0.377±0.013	0.387±0.015	0.379±0.011	0.009	[0.001, 0.016]	0.972
IFO_right	0.384±0.018	0.373±0.011	0.383±0.016	0.375±0.009	0.009	[0.001, 0.016]	0.973
ILF_left	0.389±0.017	0.381±0.011	0.388±0.016	0.383±0.011	0.008	[0.001, 0.015]	0.958
ILF_right	0.391±0.021	0.384±0.014	0.390±0.020	0.386±0.014	0.008	[0.001, 0.015]	0.959
MCP	0.456±0.020	0.447±0.014	0.456±0.020	0.448±0.013	0.008	[0.001, 0.015]	0.960
MLF_left	0.377±0.019	0.364±0.011	0.376±0.019	0.367±0.011	0.009	[0.002, 0.016]	0.974
MLF_right	0.382±0.021	0.366±0.015	0.380±0.020	0.369±0.013	0.009	[0.002, 0.017]	0.982
OR_left	0.392±0.016	0.384±0.014	0.391±0.015	0.386±0.013	0.008	[0.001, 0.015]	0.967
OR_right	0.401±0.020	0.390±0.014	0.400±0.018	0.393±0.013	0.009	[0.002, 0.016]	0.974
POPT_left	0.463±0.021	0.451±0.014	0.462±0.021	0.453±0.014	0.008	[0.001, 0.015]	0.966
POPT_right	0.464±0.019	0.449±0.015	0.462±0.018	0.452±0.014	0.009	[0.002, 0.016]	0.973
SCP_left	0.463±0.015	0.467±0.018	0.463±0.015	0.467±0.017	0.005	[-0.002, 0.013]	0.874
SCP_right	0.439±0.014	0.440±0.017	0.439±0.014	0.439±0.017	0.006	[-0.002, 0.013]	0.900
SLF_III_left	0.370±0.017	0.362±0.016	0.369±0.016	0.364±0.014	0.008	[0.001, 0.016]	0.967
SLF_III_right	0.380±0.020	0.367±0.016	0.379±0.020	0.368±0.014	0.009	[0.001, 0.016]	0.973
SLF_II_left	0.338±0.017	0.327±0.014	0.337±0.017	0.329±0.014	0.008	[0.001, 0.016]	0.969
SLF_II_right	0.333±0.020	0.324±0.015	0.332±0.019	0.325±0.014	0.008	[0.001, 0.015]	0.961
SLF_I_left	0.310±0.016	0.308±0.014	0.310±0.016	0.308±0.013	0.007	[-0.001, 0.014]	0.927
SLF_I_right	0.330±0.019	0.324±0.010	0.330±0.018	0.325±0.010	0.008	[0.000, 0.015]	0.953
STR_left	0.495±0.023	0.486±0.018	0.494±0.024	0.488±0.017	0.007	[0.000, 0.015]	0.947
STR_right	0.471±0.023	0.456±0.019	0.470±0.023	0.459±0.017	0.009	[0.002, 0.016]	0.974
UF_left	0.427±0.018	0.418±0.014	0.426±0.017	0.419±0.013	0.008	[0.001, 0.015]	0.962
UF_right	0.418±0.018	0.406±0.012	0.417±0.017	0.407±0.012	0.009	[0.002, 0.016]	0.973

[1] Units for raw and adjusted means, difference, and 95% HDI are arbitrary.

Data are presented as mean ± SD. Raw values are unadjusted sex-wise means. Adjusted values are sex-wise mean residual values after adjusting for age, education, APOE e4 carriership, and family history of Alzheimer's disease. Bayesian model outputs include the mean difference and 95% highest density interval estimates as well as P+. P+ indicates the proportion of the Bayesian samples (N = 80000) where the difference between women and men was > 0. P+ values approaching 1 indicate stronger evidence of female > male while P+ values approaching 0 indicate stronger evidence of male > female. The hierarchical model includes all tracts in a singular model and leverages partial pooling of the data, therefore the mean difference will not always be exactly equal to the difference in covariate adjusted means.

Supplemental table 12: Fiber cross-section differences between peri-menopausal women and age-matched men

	Raw means[1]		Covariate adjusted means[1]		Hierarchical Bayesian model[1]		
Tract	Females	Males	Females	Males	Mean Difference	95% HDI	P+
AF_left	0.010±0.055	0.105±0.071	0.042±0.037	0.045±0.030	-0.005	[-0.020, 0.011]	0.311
AF_right	0.009±0.054	0.104±0.069	0.041±0.033	0.044±0.036	-0.005	[-0.020, 0.011]	0.311
ATR_left	-0.013±0.067	0.081±0.081	0.022±0.046	0.016±0.042	-0.004	[-0.020, 0.011]	0.319
ATR_right	-0.009±0.064	0.088±0.091	0.027±0.046	0.021±0.050	-0.005	[-0.020, 0.011]	0.311
CA	-0.008±0.059	0.085±0.084	0.020±0.047	0.032±0.068	-0.005	[-0.020, 0.011]	0.303
CC_1	-0.014±0.080	0.057±0.086	0.014±0.060	0.004±0.068	-0.003	[-0.019, 0.013]	0.390
CC_2	-0.001±0.065	0.089±0.079	0.032±0.040	0.027±0.044	-0.004	[-0.020, 0.012]	0.342
CC_3	0.004±0.065	0.103±0.101	0.039±0.059	0.038±0.062	-0.005	[-0.021, 0.010]	0.305
CC_4	-0.016±0.061	0.071±0.065	0.011±0.053	0.019±0.043	-0.005	[-0.020, 0.011]	0.316
CC_5	-0.022±0.056	0.062±0.065	0.005±0.047	0.010±0.038	-0.005	[-0.020, 0.011]	0.312
CC_6	-0.031±0.052	0.075±0.074	0.004±0.034	0.011±0.041	-0.006	[-0.022, 0.010]	0.266
CC_7	0.010±0.069	0.139±0.094	0.052±0.053	0.063±0.054	-0.007	[-0.023, 0.009]	0.233
CG_left	-0.029±0.059	0.066±0.074	0.003±0.035	0.007±0.046	-0.005	[-0.021, 0.010]	0.301
CG_right	-0.024±0.055	0.063±0.073	0.006±0.037	0.007±0.047	-0.005	[-0.020, 0.011]	0.319
CST_left	-0.026±0.061	0.045±0.055	-0.003±0.049	0.002±0.044	-0.004	[-0.019, 0.012]	0.342
CST_right	-0.028±0.057	0.049±0.066	-0.001±0.042	0.000±0.047	-0.004	[-0.020, 0.011]	0.331
FPT_left	-0.019±0.051	0.061±0.060	0.009±0.029	0.008±0.037	-0.004	[-0.020, 0.011]	0.330
FPT_right	-0.014±0.053	0.058±0.062	0.013±0.034	0.008±0.034	-0.003	[-0.019, 0.012]	0.355
FX_left	-0.097±0.106	0.055±0.131	-0.047±0.083	-0.038±0.079	-0.009	[-0.027, 0.009]	0.217
FX_right	-0.100±0.101	0.060±0.142	-0.048±0.078	-0.037±0.087	-0.010	[-0.028, 0.010]	0.205
ICP_left	-0.018±0.066	0.022±0.044	-0.003±0.058	-0.006±0.043	-0.002	[-0.018, 0.015]	0.430
ICP_right	-0.026±0.059	0.021±0.059	-0.008±0.049	-0.013±0.056	-0.002	[-0.019, 0.014]	0.404
IFO_left	0.003±0.058	0.108±0.075	0.039±0.036	0.042±0.032	-0.006	[-0.021, 0.010]	0.281
IFO_right	0.007±0.055	0.114±0.078	0.043±0.033	0.047±0.040	-0.006	[-0.021, 0.010]	0.277
ILF_left	0.021±0.058	0.133±0.079	0.058±0.036	0.064±0.036	-0.006	[-0.022, 0.010]	0.271
ILF_right	0.020±0.059	0.131±0.083	0.057±0.038	0.061±0.042	-0.006	[-0.022, 0.010]	0.276
MCP	-0.012±0.068	0.045±0.054	0.007±0.057	0.010±0.056	-0.003	[-0.019, 0.013]	0.388
MLF_left	-0.011±0.050	0.093±0.067	0.022±0.030	0.032±0.037	-0.006	[-0.021, 0.010]	0.273
MLF_right	-0.020±0.053	0.087±0.074	0.015±0.032	0.024±0.043	-0.006	[-0.021, 0.010]	0.268
OR_left	0.007±0.063	0.119±0.093	0.045±0.051	0.050±0.048	-0.006	[-0.022, 0.009]	0.258
OR_right	-0.004±0.062	0.113±0.090	0.035±0.043	0.040±0.049	-0.007	[-0.022, 0.009]	0.250
POPT_left	-0.033±0.051	0.050±0.060	-0.005±0.034	-0.002±0.034	-0.005	[-0.021, 0.011]	0.306
POPT_right	-0.036±0.053	0.046±0.065	-0.007±0.036	-0.007±0.036	-0.005	[-0.020, 0.011]	0.317
SCP_left	-0.029±0.051	0.018±0.052	-0.009±0.039	-0.019±0.036	-0.002	[-0.019, 0.014]	0.404
SCP_right	-0.033±0.050	0.018±0.054	-0.012±0.036	-0.020±0.040	-0.003	[-0.019, 0.013]	0.388
SLF_III_left	0.009±0.059	0.109±0.095	0.044±0.044	0.044±0.058	-0.005	[-0.021, 0.010]	0.297
SLF_III_right	0.012±0.062	0.099±0.074	0.043±0.045	0.043±0.052	-0.004	[-0.020, 0.011]	0.329
SLF_II_left	0.014±0.059	0.105±0.073	0.045±0.042	0.046±0.041	-0.004	[-0.020, 0.011]	0.323
SLF_II_right	0.010±0.055	0.113±0.078	0.044±0.037	0.049±0.041	-0.005	[-0.021, 0.010]	0.288
SLF_I_left	0.004±0.050	0.087±0.066	0.032±0.036	0.035±0.042	-0.004	[-0.020, 0.011]	0.322
SLF_I_right	-0.003±0.052	0.084±0.069	0.027±0.031	0.028±0.039	-0.005	[-0.020, 0.011]	0.316
STR_left	-0.042±0.059	0.043±0.068	-0.017±0.050	-0.002±0.053	-0.006	[-0.021, 0.011]	0.290
STR_right	-0.025±0.058	0.052±0.072	0.000±0.050	0.006±0.052	-0.004	[-0.020, 0.011]	0.321
UF_left	-0.007±0.059	0.086±0.063	0.023±0.039	0.029±0.033	-0.005	[-0.020, 0.011]	0.314
UF_right	0.009±0.052	0.092±0.070	0.037±0.037	0.041±0.046	-0.004	[-0.020, 0.011]	0.342

[1] Units for raw and adjusted means, difference, and 95% HDI are arbitrary.

Data are presented as mean ± SD. Raw values are unadjusted sex-wise means. Adjusted values are sex-wise mean residual values after adjusting for age, education, intracranial volume, APOE e4 carriership, and family history of Alzheimer's disease. Bayesian model outputs include the mean difference and 95% highest density interval estimates as well as P+. P+ values approaching 1 indicate stronger evidence of female > male while P+ values approaching 0 indicate stronger evidence of male > female. The hierarchical model includes all tracts in a singular model and leverages partial pooling of the data, therefore the mean difference will not always be exactly equal to the difference in covariate adjusted means.

Supplemental table 13: FDC differences between peri-menopausal women and age-matched men

	Raw means[1]		Covariate adjusted means[1]		Hierarchical Bayesian model[1]		
Tract	Females	Males	Females	Males	Mean Difference	95% HDI	P+
AF_left	0.375±0.033	0.403±0.036	0.387±0.025	0.382±0.022	0.004	[-0.011, 0.018]	0.663
AF_right	0.398±0.033	0.427±0.038	0.409±0.027	0.407±0.026	0.003	[-0.012, 0.018]	0.638
ATR_left	0.362±0.031	0.392±0.034	0.374±0.022	0.370±0.021	0.004	[-0.011, 0.018]	0.656
ATR_right	0.364±0.028	0.394±0.037	0.375±0.022	0.372±0.028	0.004	[-0.011, 0.019]	0.664
CA	0.438±0.046	0.480±0.057	0.451±0.040	0.456±0.050	-0.001	[-0.016, 0.014]	0.463
CC_1	0.451±0.046	0.460±0.050	0.458±0.040	0.446±0.044	0.008	[-0.007, 0.024]	0.804
CC_2	0.385±0.033	0.407±0.036	0.395±0.026	0.390±0.027	0.005	[-0.009, 0.020]	0.723
CC_3	0.402±0.036	0.429±0.044	0.412±0.031	0.411±0.037	0.004	[-0.011, 0.018]	0.652
CC_4	0.454±0.044	0.474±0.039	0.462±0.041	0.460±0.034	0.005	[-0.010, 0.019]	0.689
CC_5	0.426±0.041	0.442±0.037	0.433±0.038	0.429±0.029	0.005	[-0.010, 0.020]	0.723
CC_6	0.400±0.035	0.427±0.034	0.410±0.030	0.409±0.027	0.003	[-0.011, 0.018]	0.642
CC_7	0.388±0.038	0.431±0.040	0.402±0.030	0.403±0.023	-0.001	[-0.016, 0.014]	0.469
CG_left	0.343±0.028	0.374±0.034	0.353±0.022	0.355±0.028	0.003	[-0.012, 0.018]	0.631
CG_right	0.358±0.028	0.384±0.032	0.367±0.023	0.367±0.024	0.004	[-0.011, 0.019]	0.676
CST_left	0.543±0.046	0.573±0.040	0.554±0.040	0.552±0.035	0.001	[-0.014, 0.016]	0.550
CST_right	0.510±0.040	0.538±0.039	0.520±0.035	0.519±0.036	0.001	[-0.014, 0.016]	0.561
FPT_left	0.469±0.033	0.502±0.037	0.482±0.026	0.479±0.029	0.002	[-0.013, 0.016]	0.568
FPT_right	0.460±0.031	0.489±0.038	0.471±0.025	0.468±0.030	0.003	[-0.012, 0.018]	0.622
FX_left	0.466±0.052	0.516±0.055	0.476±0.050	0.497±0.051	-0.006	[-0.022, 0.011]	0.292
FX_right	0.481±0.054	0.527±0.055	0.492±0.051	0.506±0.051	-0.004	[-0.019, 0.012]	0.361
ICP_left	0.412±0.039	0.423±0.021	0.417±0.036	0.413±0.022	0.008	[-0.008, 0.023]	0.793
ICP_right	0.373±0.030	0.380±0.026	0.379±0.027	0.370±0.025	0.009	[-0.006, 0.025]	0.833
IFO_left	0.388±0.030	0.418±0.034	0.400±0.022	0.397±0.016	0.003	[-0.012, 0.018]	0.626
IFO_right	0.385±0.031	0.414±0.033	0.396±0.024	0.393±0.018	0.003	[-0.012, 0.017]	0.625
ILF_left	0.397±0.030	0.435±0.037	0.410±0.024	0.410±0.020	0.001	[-0.014, 0.016]	0.539
ILF_right	0.397±0.036	0.436±0.043	0.411±0.029	0.409±0.026	0.001	[-0.014, 0.016]	0.533
MCP	0.456±0.046	0.472±0.030	0.463±0.041	0.458±0.031	0.006	[-0.009, 0.021]	0.735
MLF_left	0.375±0.033	0.402±0.030	0.385±0.028	0.384±0.022	0.004	[-0.011, 0.019]	0.663
MLF_right	0.376±0.034	0.402±0.035	0.385±0.029	0.385±0.026	0.004	[-0.011, 0.018]	0.653
OR_left	0.392±0.032	0.428±0.041	0.405±0.026	0.404±0.024	0.001	[-0.014, 0.016]	0.536
OR_right	0.397±0.035	0.433±0.037	0.410±0.028	0.408±0.021	0.001	[-0.014, 0.016]	0.536
POPT_left	0.450±0.037	0.475±0.033	0.460±0.031	0.456±0.025	0.003	[-0.012, 0.018]	0.640
POPT_right	0.449±0.035	0.471±0.034	0.458±0.030	0.455±0.027	0.003	[-0.011, 0.018]	0.648
SCP_left	0.450±0.033	0.476±0.028	0.460±0.027	0.456±0.025	0.003	[-0.011, 0.018]	0.642
SCP_right	0.425±0.030	0.449±0.029	0.435±0.024	0.430±0.024	0.004	[-0.010, 0.019]	0.685
SLF_III_left	0.376±0.034	0.408±0.047	0.389±0.028	0.385±0.034	0.003	[-0.012, 0.018]	0.622
SLF_III_right	0.388±0.037	0.409±0.042	0.397±0.032	0.392±0.034	0.005	[-0.010, 0.020]	0.707
SLF_II_left	0.347±0.033	0.368±0.032	0.356±0.026	0.351±0.027	0.006	[-0.009, 0.021]	0.745
SLF_II_right	0.340±0.032	0.368±0.038	0.350±0.028	0.350±0.030	0.004	[-0.011, 0.019]	0.663
SLF_I_left	0.315±0.026	0.341±0.030	0.323±0.024	0.325±0.024	0.005	[-0.010, 0.020]	0.701
SLF_I_right	0.333±0.028	0.358±0.027	0.342±0.023	0.342±0.021	0.005	[-0.010, 0.019]	0.695
STR_left	0.478±0.045	0.508±0.044	0.489±0.041	0.488±0.037	0.001	[-0.014, 0.016]	0.554
STR_right	0.461±0.045	0.480±0.044	0.468±0.041	0.466±0.040	0.004	[-0.011, 0.019]	0.673
UF_left	0.424±0.034	0.456±0.031	0.435±0.027	0.436±0.020	0.002	[-0.013, 0.017]	0.585
UF_right	0.421±0.033	0.445±0.036	0.431±0.028	0.428±0.027	0.004	[-0.011, 0.019]	0.670

[1] Units for raw and adjusted means, difference, and 95% HDI are arbitrary.

Data are presented as mean ± SD. Raw values are unadjusted sex-wise means. Adjusted values are sex-wise mean residual values after adjusting for age, education, intracranial volume, APOE e4 carriership, and family history of Alzheimer's disease. Bayesian model outputs include the mean difference and 95% highest density interval estimates as well as P+. P+ values approaching 1 indicate stronger evidence of female > male while P+ values approaching 0 indicate stronger evidence of male > female. The hierarchical model includes all tracts in a singular model and leverages partial pooling of the data, therefore the mean difference will not always be exactly equal to the difference in covariate adjusted means.

Supplemental table 14: Fractional anisotropy differences between peri-menopausal women and age-matched men

	Raw means[1]		Covariate adjusted means[1]		Hierarchical Bayesian model[1]		
Tract	Females	Males	Females	Males	Mean Difference	95% HDI	P+
AF_left	0.388±0.016	0.389±0.013	0.389±0.015	0.387±0.012	0.000	[-0.006, 0.006]	0.468
AF_right	0.388±0.016	0.387±0.012	0.389±0.015	0.385±0.011	0.000	[-0.006, 0.006]	0.476
ATR_left	0.363±0.012	0.370±0.011	0.363±0.011	0.368±0.012	-0.003	[-0.009, 0.003]	0.245
ATR_right	0.345±0.011	0.348±0.013	0.345±0.011	0.347±0.013	-0.001	[-0.007, 0.005]	0.387
CA	0.388±0.044	0.407±0.054	0.387±0.043	0.408±0.053	-0.009	[-0.017, -0.001]	0.025
CC_1	0.436±0.018	0.442±0.021	0.436±0.018	0.441±0.021	-0.003	[-0.009, 0.003]	0.220
CC_2	0.410±0.020	0.412±0.014	0.411±0.020	0.412±0.014	-0.002	[-0.008, 0.004]	0.309
CC_3	0.400±0.015	0.404±0.015	0.401±0.015	0.403±0.015	-0.002	[-0.008, 0.004]	0.258
CC_4	0.451±0.016	0.447±0.014	0.451±0.015	0.447±0.014	0.000	[-0.007, 0.006]	0.466
CC_5	0.451±0.017	0.447±0.016	0.450±0.016	0.448±0.015	-0.001	[-0.008, 0.005]	0.362
CC_6	0.478±0.012	0.480±0.017	0.478±0.012	0.479±0.017	-0.002	[-0.008, 0.004]	0.318
CC_7	0.492±0.019	0.494±0.023	0.491±0.018	0.495±0.022	-0.003	[-0.010, 0.003]	0.196
CG_left	0.409±0.018	0.421±0.016	0.411±0.017	0.418±0.014	-0.004	[-0.010, 0.003]	0.187
CG_right	0.401±0.017	0.411±0.018	0.403±0.017	0.408±0.016	-0.003	[-0.009, 0.003]	0.228
CST_left	0.496±0.018	0.497±0.013	0.498±0.016	0.494±0.011	0.000	[-0.007, 0.006]	0.481
CST_right	0.490±0.016	0.492±0.012	0.490±0.014	0.491±0.012	-0.001	[-0.007, 0.006]	0.431
FPT_left	0.459±0.015	0.468±0.013	0.461±0.013	0.465±0.012	-0.003	[-0.009, 0.003]	0.234
FPT_right	0.451±0.015	0.458±0.015	0.452±0.013	0.456±0.015	-0.002	[-0.008, 0.004]	0.305
FX_left	0.383±0.054	0.384±0.046	0.381±0.048	0.388±0.039	-0.006	[-0.013, 0.001]	0.091
FX_right	0.379±0.050	0.379±0.044	0.377±0.046	0.381±0.042	-0.006	[-0.012, 0.001]	0.093
ICP_left	0.416±0.015	0.423±0.018	0.417±0.014	0.422±0.019	-0.003	[-0.009, 0.003]	0.223
ICP_right	0.423±0.016	0.425±0.018	0.423±0.015	0.425±0.018	-0.002	[-0.008, 0.004]	0.333
IFO_left	0.420±0.013	0.421±0.011	0.421±0.012	0.420±0.012	-0.001	[-0.007, 0.005]	0.407
IFO_right	0.427±0.014	0.427±0.011	0.428±0.013	0.427±0.010	0.000	[-0.006, 0.006]	0.461
ILF_left	0.414±0.016	0.413±0.014	0.415±0.015	0.412±0.015	0.000	[-0.006, 0.006]	0.509
ILF_right	0.413±0.017	0.410±0.013	0.413±0.016	0.409±0.012	0.001	[-0.005, 0.007]	0.608
MCP	0.480±0.013	0.483±0.012	0.480±0.012	0.483±0.012	-0.003	[-0.009, 0.003]	0.250
MLF_left	0.385±0.013	0.383±0.013	0.385±0.012	0.382±0.013	0.001	[-0.005, 0.007]	0.599
MLF_right	0.375±0.014	0.376±0.015	0.376±0.013	0.375±0.015	0.000	[-0.006, 0.006]	0.515
OR_left	0.419±0.017	0.421±0.016	0.419±0.016	0.421±0.016	-0.001	[-0.007, 0.005]	0.418
OR_right	0.428±0.016	0.431±0.013	0.429±0.014	0.430±0.014	-0.001	[-0.007, 0.005]	0.431
POPT_left	0.453±0.015	0.455±0.012	0.454±0.013	0.454±0.011	-0.001	[-0.007, 0.005]	0.422
POPT_right	0.459±0.013	0.464±0.013	0.460±0.013	0.462±0.012	-0.001	[-0.007, 0.005]	0.370
SCP_left	0.429±0.012	0.439±0.013	0.430±0.012	0.437±0.013	-0.004	[-0.010, 0.002]	0.158
SCP_right	0.409±0.012	0.420±0.017	0.411±0.012	0.417±0.014	-0.004	[-0.010, 0.003]	0.172
SLF_III_left	0.396±0.013	0.398±0.013	0.397±0.013	0.397±0.012	-0.001	[-0.007, 0.005]	0.348
SLF_III_right	0.387±0.013	0.389±0.013	0.387±0.013	0.387±0.012	-0.002	[-0.008, 0.004]	0.311
SLF_II_left	0.352±0.017	0.345±0.015	0.353±0.017	0.345±0.015	0.002	[-0.004, 0.009]	0.706
SLF_II_right	0.359±0.017	0.356±0.012	0.360±0.016	0.355±0.012	0.001	[-0.005, 0.007]	0.585
SLF_I_left	0.377±0.014	0.376±0.013	0.378±0.013	0.374±0.012	0.001	[-0.006, 0.007]	0.556
SLF_I_right	0.375±0.015	0.374±0.012	0.376±0.014	0.373±0.012	0.001	[-0.006, 0.007]	0.564
STR_left	0.396±0.018	0.399±0.015	0.397±0.017	0.398±0.014	0.000	[-0.006, 0.006]	0.465
STR_right	0.399±0.018	0.402±0.016	0.400±0.017	0.401±0.016	0.000	[-0.006, 0.006]	0.466
UF_left	0.365±0.012	0.373±0.016	0.366±0.012	0.371±0.016	-0.003	[-0.009, 0.003]	0.218
UF_right	0.355±0.012	0.358±0.013	0.356±0.011	0.357±0.013	-0.001	[-0.007, 0.005]	0.364

[1] Units for raw and adjusted means, difference, and 95% HDI are arbitrary.

Data are presented as mean ± SD. Raw values are unadjusted sex-wise means. Adjusted values are sex-wise mean residual values after adjusting for age, education, APOE e4 carriership, and family history of Alzheimer's disease. Bayesian model outputs include the mean difference and 95% highest density interval estimates as well as P+. P+ indicates the proportion of the Bayesian samples (N = 80000) where the difference between women and men was > 0. P+ values approaching 1 indicate stronger evidence of female > male while P+ values approaching 0 indicate stronger evidence of male > female. The hierarchical model includes all tracts in a singular model and leverages partial pooling of the data, therefore the mean difference will not always be exactly equal to the difference in covariate adjusted means.

Supplemental table 15: Mean diffusivity differences between peri-menopausal women and age-matched men

	Raw means[1]		Covariate adjusted means[1]		Hierarchical Bayesian model[1]		
Tract	Females	Males	Females	Males	Mean Difference	95% HDI	P+
AF_left	7.132±0.206	7.071±0.179	7.123±0.197	7.088±0.185	0.029	[-0.073, 0.129]	0.680
AF_right	7.172±0.201	7.116±0.197	7.167±0.199	7.125±0.193	0.029	[-0.071, 0.130]	0.683
ATR_left	7.552±0.221	7.510±0.202	7.548±0.208	7.516±0.210	0.029	[-0.071, 0.130]	0.678
ATR_right	7.818±0.231	7.827±0.254	7.824±0.226	7.816±0.232	0.027	[-0.072, 0.128]	0.670
CA	8.156±0.328	7.948±0.291	8.153±0.323	7.954±0.277	0.035	[-0.067, 0.136]	0.708
CC_1	8.130±0.295	7.991±0.305	8.128±0.288	7.995±0.314	0.032	[-0.069, 0.133]	0.696
CC_2	8.490±0.427	8.450±0.260	8.492±0.422	8.446±0.256	0.029	[-0.071, 0.130]	0.680
CC_3	8.782±0.391	8.816±0.384	8.786±0.367	8.808±0.380	0.026	[-0.074, 0.126]	0.664
CC_4	8.385±0.328	8.421±0.319	8.388±0.299	8.415±0.317	0.026	[-0.074, 0.127]	0.666
CC_5	8.804±0.409	8.840±0.368	8.825±0.361	8.802±0.321	0.027	[-0.071, 0.130]	0.669
CC_6	7.903±0.233	7.853±0.283	7.907±0.225	7.845±0.268	0.029	[-0.071, 0.130]	0.683
CC_7	9.053±0.546	9.382±0.827	9.105±0.532	9.284±0.747	0.016	[-0.090, 0.119]	0.606
CG_left	7.264±0.200	7.158±0.219	7.250±0.197	7.183±0.208	0.031	[-0.071, 0.131]	0.688
CG_right	7.222±0.223	7.148±0.255	7.212±0.221	7.166±0.244	0.030	[-0.071, 0.131]	0.685
CST_left	6.931±0.159	6.934±0.118	6.922±0.150	6.950±0.123	0.027	[-0.074, 0.127]	0.668
CST_right	7.092±0.152	7.028±0.158	7.087±0.151	7.037±0.159	0.030	[-0.069, 0.132]	0.683
FPT_left	7.118±0.177	7.087±0.133	7.106±0.167	7.110±0.134	0.028	[-0.073, 0.128]	0.675
FPT_right	7.312±0.193	7.261±0.153	7.306±0.190	7.272±0.157	0.029	[-0.071, 0.130]	0.679
FX_left	16.218±2.083	16.426±1.960	16.313±1.813	16.249±1.653	0.026	[-0.091, 0.143]	0.646
FX_right	15.974±1.907	16.199±1.907	16.062±1.636	16.037±1.776	0.021	[-0.093, 0.140]	0.624
ICP_left	7.110±0.185	7.024±0.176	7.104±0.182	7.034±0.181	0.030	[-0.070, 0.131]	0.687
ICP_right	6.864±0.204	6.790±0.211	6.865±0.204	6.788±0.208	0.030	[-0.071, 0.131]	0.685
IFO_left	7.838±0.218	7.776±0.242	7.835±0.216	7.780±0.240	0.029	[-0.069, 0.132]	0.682
IFO_right	7.827±0.215	7.871±0.218	7.840±0.210	7.847±0.197	0.026	[-0.075, 0.126]	0.664
ILF_left	7.811±0.245	7.812±0.278	7.809±0.246	7.815±0.268	0.027	[-0.072, 0.129]	0.672
ILF_right	7.663±0.226	7.767±0.195	7.672±0.218	7.749±0.191	0.023	[-0.077, 0.124]	0.650
MCP	6.887±0.163	6.843±0.156	6.885±0.160	6.846±0.149	0.028	[-0.073, 0.129]	0.678
MLF_left	7.587±0.198	7.545±0.226	7.580±0.186	7.559±0.228	0.028	[-0.072, 0.128]	0.677
MLF_right	7.391±0.202	7.319±0.232	7.384±0.199	7.332±0.217	0.030	[-0.071, 0.129]	0.684
OR_left	8.085±0.266	8.232±0.448	8.109±0.271	8.188±0.392	0.023	[-0.080, 0.122]	0.647
OR_right	7.897±0.269	7.985±0.318	7.908±0.254	7.964±0.307	0.024	[-0.076, 0.126]	0.655
POPT_left	7.621±0.167	7.645±0.175	7.616±0.165	7.654±0.170	0.026	[-0.075, 0.126]	0.666
POPT_right	7.618±0.152	7.572±0.214	7.613±0.154	7.582±0.203	0.029	[-0.072, 0.129]	0.679
SCP_left	8.003±0.229	8.091±0.248	8.007±0.225	8.085±0.249	0.024	[-0.075, 0.126]	0.653
SCP_right	8.297±0.265	8.268±0.250	8.293±0.253	8.275±0.248	0.028	[-0.074, 0.128]	0.675
SLF_III_left	7.091±0.220	7.037±0.192	7.083±0.216	7.052±0.189	0.029	[-0.070, 0.131]	0.680
SLF_III_right	7.323±0.231	7.263±0.222	7.319±0.233	7.270±0.214	0.030	[-0.072, 0.129]	0.684
SLF_II_left	7.090±0.198	7.084±0.212	7.081±0.192	7.101±0.213	0.027	[-0.074, 0.127]	0.670
SLF_II_right	7.203±0.206	7.158±0.223	7.196±0.204	7.171±0.212	0.029	[-0.070, 0.131]	0.681
SLF_I_left	7.286±0.240	7.244±0.236	7.270±0.234	7.274±0.223	0.028	[-0.072, 0.129]	0.677
SLF_I_right	7.381±0.224	7.319±0.242	7.372±0.218	7.336±0.235	0.030	[-0.071, 0.130]	0.684
STR_left	7.020±0.199	6.985±0.172	7.015±0.196	6.993±0.170	0.029	[-0.071, 0.130]	0.679
STR_right	7.023±0.177	6.933±0.194	7.016±0.173	6.945±0.190	0.031	[-0.069, 0.133]	0.691
UF_left	7.665±0.228	7.459±0.184	7.649±0.220	7.489±0.200	0.034	[-0.068, 0.134]	0.703
UF_right	7.874±0.212	7.773±0.153	7.865±0.206	7.790±0.158	0.030	[-0.070, 0.131]	0.686

[1] Units for raw and adjusted means, difference, and 95% HDI are 10^{-4} mm²/s.

Data are presented as mean ± SD. Raw values are unadjusted sex-wise means. Adjusted values are sex-wise mean residual values after adjusting for age, education, APOE e4 carriership, and family history of Alzheimer's disease. Bayesian model outputs include the mean difference and 95% highest density interval estimates as well as P+. P+ indicates the proportion of the Bayesian samples (N = 80000) where the difference between women and men was > 0. P+ values approaching 1 indicate stronger evidence of female > male while P+ values approaching 0 indicate stronger evidence of male > female. The hierarchical model includes all tracts in a singular model and leverages partial pooling of the data, therefore the mean difference will not always be exactly equal to the difference in covariate adjusted means.

Supplemental table 16: Fiber density differences between post-menopausal women and age-matched men

	Raw means[1]		Covariate adjusted means[1]		Hierarchical Bayesian model[1]		
Tract	Females	Males	Females	Males	Mean Difference	95% HDI	P+
AF_left	0.369±0.014	0.356±0.015	0.368±0.015	0.358±0.014	0.010	[0.001, 0.018]	0.968
AF_right	0.394±0.016	0.383±0.016	0.393±0.015	0.384±0.015	0.008	[0.000, 0.017]	0.945
ATR_left	0.366±0.019	0.357±0.022	0.364±0.017	0.360±0.018	0.010	[0.002, 0.019]	0.970
ATR_right	0.363±0.017	0.360±0.020	0.362±0.017	0.362±0.017	0.007	[-0.002, 0.016]	0.894
CA	0.439±0.027	0.429±0.029	0.438±0.026	0.429±0.029	0.008	[0.000, 0.017]	0.942
CC_1	0.449±0.018	0.434±0.019	0.447±0.018	0.437±0.014	0.012	[0.003, 0.021]	0.987
CC_2	0.382±0.015	0.370±0.015	0.381±0.014	0.372±0.011	0.011	[0.003, 0.020]	0.980
CC_3	0.402±0.025	0.383±0.025	0.401±0.024	0.385±0.021	0.015	[0.006, 0.023]	0.996
CC_4	0.457±0.021	0.438±0.019	0.456±0.020	0.440±0.016	0.013	[0.004, 0.022]	0.992
CC_5	0.430±0.019	0.411±0.018	0.429±0.018	0.413±0.015	0.013	[0.004, 0.021]	0.990
CC_6	0.408±0.015	0.392±0.014	0.407±0.014	0.393±0.012	0.011	[0.003, 0.020]	0.983
CC_7	0.388±0.015	0.369±0.013	0.387±0.014	0.370±0.012	0.013	[0.004, 0.022]	0.991
CG_left	0.353±0.014	0.342±0.014	0.352±0.014	0.344±0.012	0.008	[0.000, 0.017]	0.937
CG_right	0.364±0.017	0.351±0.013	0.363±0.016	0.353±0.011	0.009	[0.000, 0.017]	0.948
CST_left	0.553±0.022	0.555±0.021	0.553±0.020	0.556±0.020	0.001	[-0.008, 0.010]	0.577
CST_right	0.520±0.018	0.516±0.019	0.520±0.018	0.517±0.017	0.003	[-0.006, 0.012]	0.708
FPT_left	0.476±0.020	0.474±0.017	0.475±0.019	0.476±0.014	0.004	[-0.004, 0.013]	0.793
FPT_right	0.468±0.019	0.464±0.019	0.467±0.019	0.466±0.016	0.005	[-0.004, 0.013]	0.809
FX_left	0.513±0.054	0.462±0.067	0.507±0.051	0.470±0.061	0.034	[0.024, 0.043]	1.000
FX_right	0.517±0.060	0.470±0.064	0.510±0.054	0.480±0.050	0.036	[0.026, 0.045]	1.000
ICP_left	0.417±0.019	0.411±0.019	0.416±0.018	0.412±0.018	0.006	[-0.003, 0.014]	0.855
ICP_right	0.382±0.018	0.374±0.014	0.381±0.017	0.375±0.013	0.008	[0.000, 0.017]	0.941
IFO_left	0.387±0.015	0.373±0.018	0.385±0.015	0.375±0.014	0.011	[0.003, 0.020]	0.983
IFO_right	0.385±0.015	0.371±0.013	0.384±0.014	0.372±0.012	0.011	[0.002, 0.019]	0.976
ILF_left	0.388±0.017	0.375±0.019	0.387±0.017	0.376±0.017	0.010	[0.001, 0.018]	0.969
ILF_right	0.393±0.016	0.385±0.021	0.392±0.015	0.386±0.021	0.007	[-0.002, 0.015]	0.909
MCP	0.455±0.019	0.444±0.017	0.454±0.018	0.446±0.016	0.007	[-0.001, 0.016]	0.917
MLF_left	0.376±0.011	0.362±0.014	0.375±0.011	0.363±0.012	0.010	[0.001, 0.019]	0.969
MLF_right	0.382±0.016	0.365±0.014	0.381±0.015	0.367±0.013	0.012	[0.003, 0.020]	0.985
OR_left	0.397±0.015	0.384±0.014	0.396±0.016	0.385±0.012	0.010	[0.001, 0.018]	0.963
OR_right	0.408±0.016	0.390±0.015	0.407±0.016	0.391±0.013	0.012	[0.003, 0.020]	0.985
POPT_left	0.464±0.013	0.454±0.014	0.463±0.012	0.455±0.011	0.007	[-0.002, 0.015]	0.897
POPT_right	0.463±0.016	0.455±0.015	0.462±0.016	0.456±0.013	0.006	[-0.003, 0.014]	0.856
SCP_left	0.469±0.018	0.474±0.014	0.469±0.017	0.474±0.014	0.000	[-0.009, 0.008]	0.467
SCP_right	0.444±0.017	0.446±0.015	0.443±0.016	0.447±0.015	0.001	[-0.008, 0.010]	0.578
SLF_III_left	0.370±0.018	0.358±0.020	0.368±0.018	0.361±0.016	0.011	[0.002, 0.019]	0.974
SLF_III_right	0.373±0.021	0.366±0.019	0.372±0.019	0.368±0.017	0.007	[-0.002, 0.016]	0.906
SLF_II_left	0.335±0.019	0.326±0.019	0.334±0.018	0.328±0.015	0.009	[0.000, 0.018]	0.951
SLF_II_right	0.333±0.016	0.322±0.015	0.332±0.016	0.323±0.013	0.009	[0.001, 0.018]	0.956
SLF_I_left	0.310±0.015	0.304±0.012	0.309±0.014	0.306±0.009	0.007	[-0.002, 0.016]	0.906
SLF_I_right	0.331±0.015	0.322±0.012	0.330±0.014	0.324±0.010	0.008	[-0.001, 0.016]	0.926
STR_left	0.496±0.023	0.491±0.020	0.496±0.022	0.491±0.020	0.004	[-0.005, 0.013]	0.763
STR_right	0.468±0.021	0.464±0.020	0.468±0.021	0.464±0.019	0.003	[-0.005, 0.012]	0.732
UF_left	0.421±0.018	0.412±0.023	0.420±0.017	0.414±0.021	0.008	[-0.001, 0.016]	0.929
UF_right	0.411±0.020	0.402±0.020	0.410±0.020	0.404±0.018	0.009	[0.000, 0.017]	0.945

[1] Units for raw and adjusted means, difference, and 95% HDI are arbitrary.

Data are presented as mean ± SD. Raw values are unadjusted sex-wise means. Adjusted values are sex-wise mean residual values after adjusting for age, education, APOE e4 carriership, and family history of Alzheimer's disease. Bayesian model outputs include the mean difference and 95% highest density interval estimates as well as P+. P+ indicates the proportion of the Bayesian samples (N = 80000) where the difference between women and men was > 0. P+ values approaching 1 indicate stronger evidence of female > male while P+ values approaching 0 indicate stronger evidence of male > female. The hierarchical model includes all tracts in a singular model and leverages partial pooling of the data, therefore the mean difference will not always be exactly equal to the difference in covariate adjusted means.

Supplemental table 17: Fiber cross-section differences between post-menopausal women and age-matched men

	Raw means[1]		Covariate adjusted means[1]		Hierarchical Bayesian model[1]		
Tract	Females	Males	Females	Males	Mean Difference	95% HDI	P+
AF_left	-0.005±0.057	0.065±0.075	0.027±0.036	0.018±0.030	0.014	[-0.003, 0.031]	0.900
AF_right	-0.005±0.053	0.070±0.075	0.027±0.031	0.021±0.034	0.013	[-0.004, 0.031]	0.889
ATR_left	-0.034±0.064	0.054±0.088	0.003±0.051	0.000±0.030	0.010	[-0.008, 0.028]	0.824
ATR_right	-0.032±0.065	0.060±0.078	0.003±0.047	0.008±0.036	0.010	[-0.007, 0.029]	0.828
CA	-0.027±0.058	0.042±0.080	0.003±0.042	-0.003±0.057	0.012	[-0.006, 0.030]	0.869
CC_1	-0.044±0.055	0.030±0.077	-0.017±0.039	-0.010±0.045	0.009	[-0.010, 0.028]	0.783
CC_2	-0.015±0.060	0.063±0.085	0.018±0.042	0.014±0.035	0.012	[-0.006, 0.029]	0.857
CC_3	0.009±0.079	0.063±0.106	0.044±0.053	0.010±0.062	0.018	[-0.002, 0.036]	0.938
CC_4	-0.005±0.060	0.051±0.097	0.022±0.051	0.011±0.051	0.014	[-0.003, 0.032]	0.904
CC_5	-0.015±0.068	0.039±0.071	0.011±0.049	0.000±0.036	0.013	[-0.004, 0.031]	0.892
CC_6	-0.017±0.054	0.037±0.067	0.010±0.032	-0.004±0.041	0.014	[-0.003, 0.032]	0.909
CC_7	0.037±0.073	0.094±0.094	0.065±0.058	0.052±0.062	0.015	[-0.003, 0.033]	0.908
CG_left	-0.035±0.059	0.033±0.065	-0.007±0.040	-0.009±0.036	0.011	[-0.007, 0.029]	0.836
CG_right	-0.040±0.052	0.044±0.058	-0.011±0.032	0.000±0.037	0.010	[-0.008, 0.028]	0.806
CST_left	-0.008±0.053	0.026±0.067	0.010±0.047	-0.001±0.040	0.014	[-0.003, 0.032]	0.908
CST_right	-0.008±0.048	0.029±0.075	0.010±0.043	0.001±0.047	0.014	[-0.003, 0.031]	0.900
FPT_left	-0.013±0.049	0.032±0.075	0.009±0.038	-0.001±0.039	0.013	[-0.004, 0.030]	0.883
FPT_right	-0.012±0.048	0.038±0.080	0.012±0.038	0.002±0.048	0.013	[-0.004, 0.031]	0.889
FX_left	-0.080±0.114	0.055±0.139	-0.019±0.081	-0.036±0.086	0.012	[-0.007, 0.032]	0.850
FX_right	-0.077±0.118	0.059±0.138	-0.022±0.090	-0.024±0.092	0.012	[-0.008, 0.031]	0.840
ICP_left	-0.025±0.060	0.013±0.062	-0.005±0.040	-0.016±0.056	0.014	[-0.003, 0.032]	0.901
ICP_right	-0.020±0.066	0.019±0.054	-0.002±0.041	-0.008±0.044	0.015	[-0.004, 0.033]	0.909
IFO_left	-0.001±0.058	0.076±0.066	0.029±0.040	0.030±0.030	0.011	[-0.006, 0.029]	0.852
IFO_right	0.007±0.057	0.075±0.067	0.035±0.040	0.033±0.037	0.012	[-0.005, 0.030]	0.875
ILF_left	0.014±0.057	0.098±0.063	0.047±0.035	0.048±0.039	0.012	[-0.006, 0.030]	0.861
ILF_right	0.010±0.063	0.101±0.074	0.048±0.038	0.045±0.038	0.012	[-0.006, 0.030]	0.866
MCP	-0.009±0.060	0.044±0.061	0.012±0.040	0.013±0.049	0.013	[-0.005, 0.031]	0.885
MLF_left	-0.012±0.053	0.058±0.063	0.019±0.031	0.012±0.035	0.013	[-0.004, 0.031]	0.891
MLF_right	-0.019±0.054	0.048±0.060	0.010±0.034	0.004±0.035	0.013	[-0.004, 0.031]	0.890
OR_left	0.014±0.067	0.084±0.077	0.044±0.045	0.038±0.047	0.013	[-0.004, 0.031]	0.886
OR_right	0.012±0.068	0.063±0.086	0.038±0.056	0.023±0.057	0.015	[-0.003, 0.033]	0.912
POPT_left	-0.013±0.049	0.022±0.053	0.007±0.034	-0.007±0.035	0.015	[-0.003, 0.032]	0.910
POPT_right	-0.016±0.046	0.013±0.052	0.002±0.033	-0.014±0.036	0.015	[-0.003, 0.033]	0.914
SCP_left	-0.030±0.036	-0.006±0.059	-0.015±0.027	-0.027±0.047	0.014	[-0.004, 0.032]	0.891
SCP_right	-0.030±0.037	-0.006±0.056	-0.016±0.030	-0.026±0.043	0.014	[-0.004, 0.032]	0.894
SLF_III_left	-0.003±0.060	0.070±0.074	0.027±0.044	0.025±0.043	0.013	[-0.005, 0.030]	0.887
SLF_III_right	-0.006±0.052	0.069±0.075	0.023±0.034	0.026±0.055	0.013	[-0.004, 0.031]	0.884
SLF_II_left	0.004±0.060	0.078±0.078	0.039±0.039	0.027±0.031	0.014	[-0.003, 0.032]	0.906
SLF_II_right	-0.004±0.063	0.077±0.072	0.030±0.044	0.025±0.031	0.013	[-0.004, 0.031]	0.882
SLF_I_left	0.016±0.055	0.065±0.072	0.042±0.039	0.027±0.040	0.015	[-0.003, 0.033]	0.919
SLF_I_right	0.005±0.056	0.057±0.063	0.031±0.034	0.018±0.041	0.014	[-0.003, 0.032]	0.911
STR_left	-0.029±0.066	0.020±0.072	-0.007±0.054	-0.013±0.052	0.013	[-0.005, 0.030]	0.881
STR_right	-0.018±0.054	0.024±0.075	0.002±0.046	-0.005±0.049	0.013	[-0.004, 0.031]	0.887
UF_left	-0.025±0.051	0.052±0.059	0.005±0.035	0.007±0.026	0.010	[-0.007, 0.028]	0.831
UF_right	-0.013±0.047	0.068±0.061	0.016±0.028	0.024±0.040	0.010	[-0.007, 0.028]	0.830

[1] Units for raw and adjusted means, difference, and 95% HDI are arbitrary.

Data are presented as mean ± SD. Raw values are unadjusted sex-wise means. Adjusted values are sex-wise mean residual values after adjusting for age, education, intracranial volume, APOE e4 carriership, and family history of Alzheimer's disease. Bayesian model outputs include the mean difference and 95% highest density interval estimates as well as P+. P+ values approaching 1 indicate stronger evidence of female > male while P+ values approaching 0 indicate stronger evidence of male > female. The hierarchical model includes all tracts in a singular model and leverages partial pooling of the data, therefore the mean difference will not always be exactly equal to the difference in covariate adjusted means.

Supplemental table 18: FDC differences between post-menopausal women and age-matched men

	Raw means[1]		Covariate adjusted means[1]		Hierarchical Bayesian model[1]		
Tract	Females	Males	Females	Males	Mean Difference	95% HDI	P+
AF_left	0.372±0.030	0.382±0.033	0.380±0.026	0.370±0.022	0.011	[-0.004, 0.026]	0.888
AF_right	0.395±0.028	0.412±0.034	0.404±0.024	0.398±0.024	0.010	[-0.005, 0.025]	0.850
ATR_left	0.355±0.028	0.377±0.036	0.364±0.028	0.364±0.022	0.008	[-0.008, 0.023]	0.788
ATR_right	0.354±0.027	0.381±0.031	0.362±0.026	0.368±0.023	0.007	[-0.008, 0.022]	0.772
CA	0.427±0.035	0.447±0.054	0.439±0.030	0.429±0.046	0.010	[-0.005, 0.026]	0.866
CC_1	0.433±0.025	0.445±0.046	0.438±0.025	0.439±0.030	0.008	[-0.008, 0.023]	0.788
CC_2	0.380±0.027	0.394±0.037	0.387±0.025	0.383±0.024	0.010	[-0.005, 0.025]	0.860
CC_3	0.409±0.047	0.407±0.042	0.417±0.040	0.395±0.036	0.014	[-0.002, 0.030]	0.928
CC_4	0.459±0.039	0.463±0.049	0.465±0.035	0.454±0.037	0.013	[-0.003, 0.028]	0.909
CC_5	0.426±0.038	0.428±0.033	0.431±0.034	0.421±0.023	0.012	[-0.003, 0.027]	0.901
CC_6	0.404±0.030	0.406±0.029	0.410±0.025	0.397±0.022	0.013	[-0.002, 0.028]	0.914
CC_7	0.402±0.038	0.402±0.038	0.408±0.034	0.394±0.028	0.015	[-0.001, 0.030]	0.934
CG_left	0.343±0.024	0.354±0.026	0.349±0.022	0.346±0.020	0.010	[-0.005, 0.025]	0.863
CG_right	0.352±0.023	0.368±0.024	0.358±0.022	0.359±0.019	0.009	[-0.006, 0.024]	0.831
CST_left	0.551±0.044	0.569±0.043	0.560±0.040	0.556±0.028	0.009	[-0.006, 0.025]	0.828
CST_right	0.518±0.035	0.530±0.043	0.524±0.034	0.521±0.033	0.009	[-0.006, 0.024]	0.830
FPT_left	0.472±0.035	0.488±0.043	0.480±0.033	0.476±0.028	0.008	[-0.007, 0.023]	0.814
FPT_right	0.464±0.032	0.480±0.046	0.472±0.032	0.469±0.035	0.009	[-0.006, 0.024]	0.820
FX_left	0.467±0.036	0.475±0.053	0.470±0.034	0.469±0.051	0.009	[-0.006, 0.025]	0.845
FX_right	0.470±0.042	0.485±0.052	0.477±0.040	0.475±0.041	0.009	[-0.006, 0.025]	0.841
ICP_left	0.409±0.030	0.420±0.030	0.414±0.024	0.412±0.028	0.009	[-0.007, 0.023]	0.819
ICP_right	0.377±0.033	0.382±0.022	0.381±0.025	0.376±0.018	0.009	[-0.006, 0.024]	0.825
IFO_left	0.384±0.029	0.398±0.027	0.391±0.026	0.388±0.018	0.010	[-0.005, 0.025]	0.858
IFO_right	0.384±0.027	0.394±0.025	0.390±0.025	0.385±0.018	0.011	[-0.004, 0.026]	0.887
ILF_left	0.391±0.030	0.411±0.032	0.400±0.025	0.397±0.025	0.010	[-0.005, 0.025]	0.858
ILF_right	0.393±0.034	0.423±0.039	0.408±0.024	0.401±0.030	0.009	[-0.006, 0.025]	0.836
MCP	0.456±0.039	0.470±0.037	0.463±0.032	0.459±0.030	0.007	[-0.009, 0.022]	0.767
MLF_left	0.373±0.022	0.381±0.023	0.380±0.018	0.371±0.016	0.011	[-0.003, 0.027]	0.889
MLF_right	0.376±0.029	0.382±0.024	0.382±0.024	0.373±0.017	0.012	[-0.003, 0.027]	0.895
OR_left	0.399±0.032	0.411±0.029	0.406±0.028	0.400±0.021	0.011	[-0.004, 0.026]	0.888
OR_right	0.408±0.034	0.411±0.034	0.414±0.031	0.401±0.025	0.013	[-0.002, 0.029]	0.918
POPT_left	0.459±0.027	0.463±0.027	0.464±0.023	0.455±0.021	0.011	[-0.004, 0.026]	0.881
POPT_right	0.456±0.031	0.460±0.027	0.461±0.028	0.453±0.020	0.011	[-0.004, 0.026]	0.883
SCP_left	0.457±0.030	0.473±0.029	0.464±0.027	0.463±0.024	0.008	[-0.007, 0.023]	0.801
SCP_right	0.433±0.029	0.445±0.027	0.439±0.027	0.436±0.021	0.008	[-0.007, 0.023]	0.809
SLF_III_left	0.373±0.031	0.386±0.034	0.380±0.029	0.375±0.026	0.009	[-0.006, 0.024]	0.843
SLF_III_right	0.374±0.030	0.395±0.034	0.382±0.027	0.383±0.031	0.008	[-0.007, 0.023]	0.796
SLF_II_left	0.341±0.030	0.356±0.029	0.349±0.026	0.344±0.022	0.010	[-0.005, 0.025]	0.855
SLF_II_right	0.336±0.030	0.351±0.027	0.344±0.027	0.340±0.022	0.010	[-0.005, 0.025]	0.856
SLF_I_left	0.319±0.023	0.328±0.029	0.324±0.021	0.320±0.020	0.011	[-0.004, 0.026]	0.877
SLF_I_right	0.337±0.026	0.345±0.026	0.343±0.022	0.336±0.020	0.011	[-0.004, 0.026]	0.885
STR_left	0.486±0.047	0.500±0.039	0.495±0.042	0.487±0.030	0.010	[-0.005, 0.025]	0.866
STR_right	0.462±0.039	0.474±0.043	0.468±0.037	0.464±0.034	0.010	[-0.005, 0.025]	0.857
UF_left	0.411±0.026	0.432±0.032	0.419±0.024	0.420±0.026	0.008	[-0.007, 0.023]	0.799
UF_right	0.406±0.026	0.429±0.033	0.414±0.024	0.416±0.026	0.008	[-0.008, 0.023]	0.794

[1] Units for raw and adjusted means, difference, and 95% HDI are arbitrary.

Data are presented as mean ± SD. Raw values are unadjusted sex-wise means. Adjusted values are sex-wise mean residual values after adjusting for age, education, intracranial volume, APOE e4 carriership, and family history of Alzheimer's disease. Bayesian model outputs include the mean difference and 95% highest density interval estimates as well as P+. P+ values approaching 1 indicate stronger evidence of female > male while P+ values approaching 0 indicate stronger evidence of male > female. The hierarchical model includes all tracts in a singular model and leverages partial pooling of the data, therefore the mean difference will not always be exactly equal to the difference in covariate adjusted means.

Supplemental table 19: Fractional anisotropy differences between post-menopausal women and age-matched men

Tract	Raw means[1]		Covariate adjusted means[1]		Hierarchical Bayesian model[1]		
	Females	Males	Females	Males	Mean Difference	95% HDI	P+
AF_left	0.386±0.015	0.379±0.018	0.385±0.015	0.380±0.017	0.003	[-0.006, 0.011]	0.691
AF_right	0.385±0.013	0.382±0.017	0.384±0.013	0.383±0.015	0.000	[-0.008, 0.009]	0.533
ATR_left	0.360±0.015	0.365±0.013	0.359±0.014	0.366±0.013	-0.003	[-0.011, 0.006]	0.316
ATR_right	0.342±0.013	0.344±0.013	0.341±0.012	0.345±0.013	0.000	[-0.008, 0.009]	0.521
CA	0.394±0.043	0.386±0.058	0.395±0.043	0.385±0.057	0.005	[-0.003, 0.014]	0.837
CC_1	0.433±0.015	0.428±0.025	0.431±0.014	0.431±0.020	0.004	[-0.005, 0.012]	0.749
CC_2	0.409±0.015	0.404±0.018	0.408±0.015	0.405±0.014	0.004	[-0.004, 0.012]	0.778
CC_3	0.403±0.017	0.397±0.020	0.403±0.016	0.398±0.017	0.005	[-0.004, 0.013]	0.824
CC_4	0.448±0.017	0.438±0.017	0.448±0.017	0.438±0.013	0.006	[-0.003, 0.014]	0.863
CC_5	0.448±0.020	0.435±0.026	0.448±0.021	0.435±0.022	0.007	[-0.002, 0.015]	0.894
CC_6	0.478±0.016	0.474±0.016	0.478±0.016	0.475±0.014	0.003	[-0.006, 0.011]	0.683
CC_7	0.493±0.027	0.475±0.022	0.492±0.025	0.477±0.018	0.012	[0.003, 0.021]	0.984
CG_left	0.409±0.019	0.414±0.023	0.409±0.019	0.414±0.022	-0.005	[-0.013, 0.004]	0.191
CG_right	0.399±0.017	0.400±0.024	0.398±0.017	0.400±0.023	-0.003	[-0.012, 0.005]	0.262
CST_left	0.489±0.017	0.495±0.017	0.489±0.016	0.495±0.016	-0.005	[-0.013, 0.004]	0.178
CST_right	0.483±0.016	0.492±0.014	0.484±0.015	0.492±0.013	-0.007	[-0.016, 0.001]	0.081
FPT_left	0.456±0.015	0.465±0.015	0.456±0.015	0.466±0.014	-0.006	[-0.014, 0.003]	0.127
FPT_right	0.447±0.015	0.457±0.016	0.447±0.015	0.457±0.015	-0.007	[-0.015, 0.002]	0.098
FX_left	0.390±0.042	0.357±0.057	0.386±0.041	0.363±0.053	0.021	[0.012, 0.030]	1.000
FX_right	0.377±0.046	0.342±0.053	0.373±0.047	0.349±0.045	0.025	[0.016, 0.034]	1.000
ICP_left	0.411±0.015	0.417±0.017	0.411±0.015	0.417±0.016	-0.005	[-0.013, 0.003]	0.172
ICP_right	0.417±0.018	0.422±0.019	0.416±0.017	0.423±0.017	-0.004	[-0.012, 0.005]	0.255
IFO_left	0.418±0.015	0.410±0.016	0.417±0.015	0.411±0.014	0.005	[-0.004, 0.013]	0.821
IFO_right	0.425±0.016	0.418±0.017	0.424±0.016	0.420±0.015	0.004	[-0.004, 0.012]	0.788
ILF_left	0.409±0.016	0.401±0.021	0.408±0.016	0.402±0.018	0.005	[-0.003, 0.014]	0.849
ILF_right	0.409±0.016	0.408±0.013	0.409±0.015	0.409±0.012	0.001	[-0.008, 0.009]	0.561
MCP	0.480±0.015	0.482±0.016	0.480±0.015	0.482±0.015	-0.004	[-0.012, 0.005]	0.228
MLF_left	0.384±0.014	0.377±0.016	0.383±0.014	0.378±0.014	0.004	[-0.005, 0.012]	0.752
MLF_right	0.376±0.015	0.372±0.014	0.375±0.015	0.372±0.013	0.003	[-0.006, 0.011]	0.690
OR_left	0.423±0.017	0.413±0.014	0.422±0.017	0.415±0.013	0.005	[-0.003, 0.014]	0.839
OR_right	0.431±0.019	0.425±0.018	0.430±0.020	0.426±0.015	0.004	[-0.005, 0.012]	0.767
POPT_left	0.452±0.013	0.455±0.012	0.452±0.012	0.455±0.011	-0.003	[-0.011, 0.005]	0.278
POPT_right	0.458±0.014	0.464±0.013	0.458±0.013	0.464±0.012	-0.005	[-0.013, 0.004]	0.171
SCP_left	0.433±0.013	0.445±0.010	0.433±0.013	0.444±0.010	-0.009	[-0.017, 0.000]	0.050
SCP_right	0.413±0.015	0.428±0.015	0.413±0.015	0.428±0.014	-0.010	[-0.018, -0.001]	0.032
SLF_III_left	0.391±0.016	0.387±0.016	0.390±0.016	0.388±0.015	0.003	[-0.006, 0.011]	0.686
SLF_III_right	0.380±0.015	0.380±0.017	0.380±0.014	0.381±0.015	-0.001	[-0.009, 0.008]	0.446
SLF_II_left	0.351±0.017	0.345±0.018	0.350±0.017	0.346±0.017	0.003	[-0.006, 0.011]	0.700
SLF_II_right	0.357±0.015	0.354±0.014	0.357±0.016	0.354±0.013	0.001	[-0.007, 0.010]	0.592
SLF_I_left	0.374±0.018	0.376±0.016	0.374±0.016	0.376±0.014	-0.001	[-0.009, 0.008]	0.446
SLF_I_right	0.375±0.016	0.374±0.015	0.375±0.016	0.374±0.014	0.000	[-0.008, 0.009]	0.523
STR_left	0.394±0.020	0.398±0.018	0.395±0.020	0.396±0.016	-0.004	[-0.012, 0.005]	0.244
STR_right	0.393±0.018	0.405±0.020	0.394±0.017	0.403±0.020	-0.008	[-0.017, 0.000]	0.057
UF_left	0.359±0.015	0.358±0.021	0.358±0.015	0.360±0.020	0.000	[-0.009, 0.008]	0.498
UF_right	0.349±0.014	0.348±0.019	0.348±0.014	0.350±0.017	0.000	[-0.008, 0.009]	0.538

[1] Units for raw and adjusted means, difference, and 95% HDI are arbitrary.

Data are presented as mean ± SD. Raw values are unadjusted sex-wise means. Adjusted values are sex-wise mean residual values after adjusting for age, education, APOE e4 carriership, and family history of Alzheimer's disease. Bayesian model outputs include the mean difference and 95% highest density interval estimates as well as P+. P+ indicates the proportion of the Bayesian samples (N = 80000) where the difference between women and men was > 0. P+ values approaching 1 indicate stronger evidence of female > male while P+ values approaching 0 indicate stronger evidence of male > female. The hierarchical model includes all tracts in a singular model and leverages partial pooling of the data, therefore the mean difference will not always be exactly equal to the difference in covariate adjusted means.

Supplemental table 20: Mean diffusivity differences between post-menopausal women and age-matched men

	Raw means[1]		Covariate adjusted means[1]		Hierarchical Bayesian model[1]		
Tract	Females	Males	Females	Males	Mean Difference	95% HDI	P+
AF_left	7.141±0.193	7.204±0.203	7.145±0.198	7.199±0.189	-0.024	[-0.157, 0.110]	0.386
AF_right	7.218±0.174	7.184±0.245	7.217±0.181	7.186±0.211	-0.012	[-0.148, 0.121]	0.440
ATR_left	7.568±0.202	7.675±0.217	7.575±0.208	7.664±0.190	-0.062	[-0.195, 0.070]	0.222
ATR_right	7.840±0.183	7.944±0.210	7.847±0.187	7.934±0.161	-0.082	[-0.212, 0.054]	0.161
CA	8.128±0.398	8.253±0.395	8.146±0.380	8.227±0.380	-0.086	[-0.225, 0.049]	0.157
CC_1	8.123±0.320	8.074±0.334	8.127±0.313	8.068±0.249	-0.074	[-0.210, 0.062]	0.188
CC_2	8.397±0.244	8.554±0.265	8.402±0.228	8.547±0.225	-0.134	[-0.267, 0.001]	0.056
CC_3	8.670±0.353	8.904±0.329	8.680±0.325	8.889±0.309	-0.168	[-0.300, -0.032]	0.023
CC_4	8.365±0.345	8.639±0.318	8.378±0.328	8.620±0.276	-0.155	[-0.291, -0.021]	0.033
CC_5	8.834±0.398	9.239±0.638	8.850±0.394	9.215±0.578	-0.212	[-0.352, -0.071]	0.007
CC_6	7.912±0.242	8.072±0.295	7.923±0.237	8.054±0.262	-0.098	[-0.231, 0.034]	0.115
CC_7	9.242±0.776	9.637±0.724	9.277±0.662	9.584±0.679	-0.250	[-0.398, -0.106]	0.003
CG_left	7.298±0.181	7.253±0.252	7.294±0.191	7.259±0.218	-0.013	[-0.145, 0.122]	0.434
CG_right	7.272±0.181	7.239±0.215	7.267±0.188	7.246±0.183	-0.014	[-0.147, 0.119]	0.431
CST_left	6.967±0.129	6.987±0.120	6.970±0.126	6.983±0.116	-0.001	[-0.136, 0.133]	0.493
CST_right	7.107±0.131	7.061±0.139	7.106±0.134	7.064±0.130	0.001	[-0.134, 0.135]	0.503
FPT_left	7.140±0.123	7.131±0.169	7.137±0.128	7.135±0.157	-0.009	[-0.146, 0.121]	0.457
FPT_right	7.354±0.130	7.278±0.169	7.348±0.137	7.287±0.151	-0.010	[-0.145, 0.123]	0.445
FX_left	16.112±1.634	17.298±2.252	16.271±1.532	17.060±2.148	-0.807	[-0.998, -0.614]	0.000
FX_right	16.180±1.838	17.346±2.033	16.380±1.692	17.047±1.727	-0.820	[-1.011, -0.628]	0.000
ICP_left	7.141±0.166	7.100±0.161	7.139±0.149	7.104±0.168	0.001	[-0.134, 0.135]	0.505
ICP_right	6.873±0.162	6.819±0.161	6.876±0.147	6.814±0.167	0.023	[-0.112, 0.157]	0.609
IFO_left	7.819±0.241	7.988±0.265	7.831±0.242	7.971±0.212	-0.094	[-0.227, 0.039]	0.127
IFO_right	7.905±0.322	8.013±0.394	7.920±0.309	7.990±0.315	-0.091	[-0.226, 0.044]	0.136
ILF_left	7.824±0.283	8.006±0.299	7.841±0.282	7.980±0.216	-0.096	[-0.229, 0.038]	0.125
ILF_right	7.770±0.349	7.855±0.382	7.790±0.337	7.826±0.294	-0.074	[-0.207, 0.062]	0.185
MCP	6.857±0.133	6.768±0.156	6.855±0.120	6.771±0.162	0.030	[-0.106, 0.166]	0.635
MLF_left	7.576±0.190	7.689±0.256	7.584±0.194	7.677±0.229	-0.063	[-0.197, 0.068]	0.224
MLF_right	7.409±0.194	7.437±0.288	7.415±0.196	7.429±0.259	-0.037	[-0.170, 0.095]	0.323
OR_left	8.125±0.340	8.531±0.520	8.145±0.340	8.501±0.484	-0.163	[-0.303, -0.025]	0.026
OR_right	7.968±0.443	8.173±0.521	7.995±0.404	8.134±0.402	-0.119	[-0.259, 0.019]	0.084
POPT_left	7.667±0.172	7.747±0.168	7.673±0.172	7.737±0.163	-0.060	[-0.192, 0.075]	0.234
POPT_right	7.629±0.139	7.674±0.211	7.636±0.141	7.663±0.201	-0.050	[-0.182, 0.084]	0.269
SCP_left	8.017±0.204	8.051±0.191	8.014±0.186	8.055±0.201	-0.072	[-0.208, 0.063]	0.194
SCP_right	8.270±0.231	8.268±0.197	8.277±0.232	8.257±0.186	-0.083	[-0.216, 0.052]	0.159
SLF_III_left	7.116±0.217	7.176±0.203	7.122±0.220	7.168±0.187	-0.022	[-0.156, 0.112]	0.391
SLF_III_right	7.369±0.207	7.356±0.251	7.369±0.217	7.356±0.213	-0.024	[-0.158, 0.109]	0.383
SLF_II_left	7.104±0.166	7.155±0.240	7.108±0.170	7.149±0.228	-0.018	[-0.153, 0.112]	0.412
SLF_II_right	7.213±0.172	7.234±0.234	7.213±0.171	7.233±0.211	-0.023	[-0.155, 0.112]	0.390
SLF_I_left	7.345±0.187	7.336±0.257	7.352±0.189	7.325±0.243	-0.022	[-0.156, 0.110]	0.391
SLF_I_right	7.412±0.215	7.421±0.263	7.417±0.203	7.414±0.242	-0.033	[-0.166, 0.101]	0.343
STR_left	7.053±0.200	7.095±0.179	7.051±0.200	7.097±0.154	-0.017	[-0.149, 0.117]	0.422
STR_right	7.047±0.162	7.008±0.198	7.044±0.165	7.012±0.159	0.001	[-0.131, 0.135]	0.502
UF_left	7.686±0.195	7.624±0.200	7.688±0.192	7.620±0.187	-0.033	[-0.167, 0.101]	0.341
UF_right	7.903±0.203	7.895±0.198	7.908±0.207	7.888±0.178	-0.056	[-0.191, 0.078]	0.244

[1] Units for raw and adjusted means, difference, and 95% HDI are 10^{-4} mm²/s.

Data are presented as mean ± SD. Raw values are unadjusted sex-wise means. Adjusted values are sex-wise mean residual values after adjusting for age, education, APOE e4 carriership, and family history of Alzheimer's disease. Bayesian model outputs include the mean difference and 95% highest density interval estimates as well as P+. P+ indicates the proportion of the Bayesian samples (N = 80000) where the difference between women and men was > 0. P+ values approaching 1 indicate stronger evidence of female > male while P+ values approaching 0 indicate stronger evidence of male > female. The hierarchical model includes all tracts in a singular model and leverages partial pooling of the data, therefore the mean difference will not always be exactly equal to the difference in covariate adjusted means.
